# Gut microbiome and metabolites mediate the benefits of caloric restriction in mice after acute kidney injury

**DOI:** 10.1016/j.redox.2024.103373

**Published:** 2024-09-27

**Authors:** Xue-Xue Zhu, Xiao Fu, Xin-Yu Meng, Jia-Bao Su, Guan-Li Zheng, An-Jing Xu, Guo Chen, Yuan Zhang, Yao Liu, Xiao-Hui Hou, Hong-Bo Qiu, Qing-Yi Sun, Jin-Yi Hu, Zhuo-Lin Lv, Yao Wang, Hai-Bin Jiang, Neng Bao, Zhi-Jun Han, Qing-Bo Lu, Hai-Jian Sun

**Affiliations:** a Wuxi School of Medicine, Department of Endocrinology, Affiliated Hospital of Jiangnan University, Jiangnan University, Wuxi, 214122, China; b Department of Anesthesiology, Affiliated Hospital of Jiangnan University, Jiangnan University, Wuxi, 214122, China; c Department of Ultrasound, The Fourth Affiliated Hospital of Nanjing Medical University, Nanjing, Jiangsu, 210000, China; d Department of Cardiology, Wuxi No.2 People's Hospital (Jiangnan University Medical Center), Wuxi, Jiangsu, China; e Department of Nephrology, Affiliated Hospital of Jiangnan University, Jiangnan University, Wuxi, 214122, China; f Department of Clinical Research Center, Jiangnan University Medical Center (Wuxi No.2 People’s Hospital), Wuxi School of Medicine, Jiangnan University, Wuxi, China; g Department of Endocrinology, Affiliated Hospital of Jiangnan University, Jiangnan University, Wuxi, 214122, China; h State Key Laboratory of Natural Medicines, China Pharmaceutical University, No. 24 Tongjia Lane, Nanjing, 210009, China

**Keywords:** Caloric restriction, Microbiome, Metabolites, Acute kidney injury, Oxidative stress, Inflammation

## Abstract

The role of gut microbiome in acute kidney injury (AKI) is increasing recognized. Caloric restriction (CR) has been shown to enhance the resistance to ischemia/reperfusion injury to the kidneys in rodents. Nonetheless, it is unknown whether intestinal microbiota mediated CR protection against ischemic/reperfusion-induced injury (IRI) in the kidneys. Herein, we showed that CR ameliorated IRI-elicited renal dysfunction, oxidative stress, apoptosis, and inflammation, along with enhanced intestinal barrier function. In addition, gut microbiota depletion blocked the favorable effects of CR in AKI mice. 16S rRNA and metabolomics analysis showed that CR enriched the gut commensal *Parabacteroides goldsteinii* (*P. goldsteinii*) and upregulated the level of serum metabolite dodecafluorpentan. Intestinal colonization of *P. goldsteinii* and oral administration of dodecafluorpentan showed the similar beneficial effects as CR in AKI mice. RNA sequencing and experimental data revealed that dodecafluorpentan protected against AKI-induced renal injury by antagonizing oxidative burst and NFκB-induced NLRP3 inflammasome activation. In addition, we screened and found that Hamaudol improved renal insufficiency by boosting the growth of *P. goldsteinii*. Our results shed light on the role of intestinal microbiota *P. goldsteinii* and serum metabolites dodecafluorpentan in CR benefits to AKI.

## Highlights

• The role of intestinal microbiota-mediated caloric restriction (CR) in ischemic/reperfusion-induced injury (IRI) in the kidneys is rarely known.
• Intestinal microbiota *Parabacteroides goldsteinii* (*P. goldsteinii*) contributes to CR protection against renal damage induced by acute kidney injury (AKI).
• Serum metabolites dodecafluorpentan alleviates the renal dysfunction after IRI.
• Hamaudol is a chromone isolated from *Saposhnikovia divaricate*, exhibiting a protection against renal IRI by boosting the growth of *P. goldsteinii.*

## 1 Introduction

Acute kidney injury (AKI) manifests a rapid decline in renal function and poses a significant health burden because of its high morbidity and mortality rates [1,2]. The prevention of AKI remains a medical challenge since AKI patients typically seek treatment only after experiencing evident clinical symptoms. Therefore, early detection is crucial for providing supportive care and limiting the disease progression of AKI [3]. To data, the diagnosis of AKI currently relies heavily on conventional markers such as serum creatinine (Scr) and blood urea nitrogen (BUN). However, the sensitivity and accuracy of these markers are still under debate, making early identification of AKI challenging [4]. In light of these challenges, there is an urgent need to identify specific diagnostic markers for the timely diagnosis and effective treatment of AKI.

Clinical and animal experimental studies have established that alterations in metabolic profiles of AKI patients and rodents are associated with the worsening of kidney disease outcomes and the progression of renal fibrosis, with studies showing an increase in plasma trimethylamine N-oxide (TMAO) levels in AKI patients [5]. A newly developed global metabolomics analysis has revealed significant changes in plasma metabolome of mice after renal IRI, with 3-indoxyl sulfate is emerging as a diagnostic biomarker for AKI [6]. It is believed that the changes in metabolic profiles may provide novel potential circulating biomarker for the diagnosis and prognosis of AKI patients. A wealth of evidence has highlighted the close relationship between the intestinal microbiota and renal physiology and pathophysiology [7]. Clinically, disturbances in the abundance of *Escherichia coli, Bacteroidetes, Bifidobacterium, Salmonella, Lactobacillus, Clostridium, Ruminococcus, Rothia, Staphylococcus, Enterobacter, Faecalibacterium,* and *Lachnospiraceae* have been observed in AKI patients [8]. Oral administration of *Lactobacillus casei* CERELA (CRL) 431 significantly decreases the production of TNF-α and IL-6 in the kidneys from lipopolysaccharides (LPS)-induced AKI mice [9]. Moreover, *Enterobacteriaceae*, such as *Escherichia coli* and *Klebsiella oxytoca*, have been shown to produce d-alanine in the gut, and oral administration of d-alanine ameliorates renal injury in mice after renal IRI [10,11]. Overall, these experimental data collectively illustrate that the significant impact of the intestinal microbiota and metabolites on the progression and outcome of AKI.

Caloric restriction (CR) stands out as one of the most effective non-generic approaches for extending lifespan. Initially observed in rodents, this phenomenon has been validated across various organisms, including non-human primates [12]. CR has demonstrated remarkable efficacy in protecting the kidneys in rodent models of AKI [[13], [14], [15], [16]]. However, the precise roles of gut microbiome and metabolites in the beneficial effects of CR on renal dysfunction post-IRI have yet to be completely elucidated. In this study, we found that CR ameliorated renal IRI in AKI mice by enhancing the gut commensal *Parabacteroides goldsteinii* (*P. goldsteinii*) and upregulating the level of serum metabolite dodecafluorpentan. *P. goldsteinii* has been established to improve integrity and insulin resistance in obese mice [17], and ameliorate chronic obstructive pulmonary disease (COPD) in mice [18]. It is possible that *P. goldsteinii* may be a potential probiotic in human disorders. It is noted that the role of dodecafluorpentan in human health and diseases has not been explored. Hamaudol is a chromone isolated from *Saposhnikovia divaricate*, and this compound has a significant inhibitory effect on the activity of cyclooxygenase-1 (COX) and COX-2 [19]. Hamaudol is found to selectively inhibit the Syk and Lyn [20], and protects against atopic dermatitis [21]. However, it remains to be answered whether Hamaudol provides a protection against renal IRI in AKI mice. As such, we aimed to investigate whether *P. goldsteinii* and dodecafluorpentan contributed to CR protection against renal damage induced by AKI, and to examine whether Hamaudol attenuated AKI-triggered renal injury by regulating *P. goldsteinii* and dodecafluorpentan.

## 2 Materials and methods

### 2.1 Reagents and chemicals

Dulbecco's modified Eagle's medium (DMEM)/F12, fetal bovine serum (FBS), trypsin, and 1 % penicillin/streptomycin solution were procured from Hyclone Laboratories (South Logan, UT, USA). Western and IP Lysis Buffer and proteinase inhibitor were acquired from Beyotime Biotechnology (Shanghai, China). Commercial kits for blood urea nitrogen (BUN) and serum creatinine (SCr) were purchased from Jiancheng Bioengineering Institute (Nanjing, China). A commercial mouse cystatin C kit was obtained from Elabscience (Wuhan, China). Dihydroethidium (DHE) and Hamaudol were procured from MCE (Shanghai, China). Dodecafluorpentan was acquired from Chemical Book. The kits for the measurement of tumor necrosis factor-α (TNF-α) and interleukin-6 (IL-6) were obtained from BOSTER (Wuhan, China). A small compound library was purchased from TOPSCIENCE (Table S1, Shanghai, China). All other reagents used were of at least analytical purity.

### 2.2 Animals

Male C57BL/6 mice were sourced from the Spfbiotech (Nanjing, China) at the age of 9–10 weeks and housed in a pathogen-free environment under a standard laboratory condition with temperatures maintained between 20 and 24 °C and relative humidity between 50 and 60 %, following a 12/12 light/dark cycle. These *ad libitum* mice had unrestricted access to water and food with 3–4 animals per cage [13], except where specified otherwise. After one week of adaptation, these mice were systematically allocated to distinct dietary regimens. One group was maintained on the standard diet, while the other group underwent a calorie-restricted (CR) diet, which received only 70 % of the caloric intake of the normal diet over a period of 4 weeks as previously described [13,22]. The average food intake was approximately 3.5 g/day in *ad libitum* fed mice, determined by daily weighing of the remaining food over one week. The CR group was implemented for 4 weeks by feeding the mice 70 % of this amount daily (2.45g/day) [13]. The CR mice had unrestricted access to water with one animal per cage. To ensure equal caloric intake across all groups, the intake of food access was precisely measured daily. Despite significant weight loss due to short-term CR, no morbidity or mortality was observed solely from these diets. All experimental procedures were conducted with approval from the Jiangnan University Institutional Animal Care and Use Committee and the criteria in the Guide for the Care and Use of Laboratory Animals published by the US National Institutes of Health. After normal diet or CR for 4 weeks, the mice were subjected to ischemia-reperfusion injury (IRI) treatment [23,24]. To determine the potential role of Dodecafluorpentan or Hamaudol in renal IRI, the mice were subjected to daily oral injection of Dodecafluorpentan (30 mg/kg) or Hamaudol (30 mg/kg) for 7 days prior to renal IRI surgery. To determine the combined effect of CR and Hamaudol on renal IRI, the mice were subjected to oral injection of Hamaudol (30 mg/kg) every other day during the CR period. After 4 weeks, the mice received renal IRI surgery. Following anesthesia with isoflurane (2 % for induction and 1.5 % for maintenance) [25], an incision was created at the junction of the rib and spine to facilitate kidney access. This allowed for the clamping of the renal pedicles using microaneurysm clips for a period of 30 min. After this period, the clips were removed, mice were placed within the incubator (37 °C) until completely awake. After 24 h, the mice were anesthetized with 5 % isoflurane, anesthesia was confirmed via tail pinch, and then sacrificed by cervical dislocation. The kidneys, colon tissues and blood samples were collected and stored at −80 °C. Serum samples were obtained through centrifugation at 1500 rpm for 20 min. In addition, the remaining renal and colonic tissues were fixed in 4 % paraformaldehyde (PFA) for histological analysis.

### 2.3 Study population

This study was adhered to the ethical principles outlined in the 1975 Declaration of Helsinki and received prior approval from the Ethics Committee of Jiangnan University (LS2024240). Upon obtaining the written informed consent, patients were asked to complete a brief questionnaire and their medical records were reviewed anonymously. Clinical data encompassed demographic details (age, gender), admission diagnosis, comorbidities, vital signs, body weight, and serum levels of serum creatinine (Scr) and blood urea nitrogen (BUN) upon admission. We utilized the Scr and urine output parameters outlined in the Kidney Disease: Improving Global Outcomes (KDIGO) criteria to define AKI [26]. The baseline Scr level was determined as the lowest value within the preceding three months before surgery. AKI was identified by either an absolute increase in Scr of at least 0.3 mg/dl (≥26.5 μmol/l) within 48 h post-surgery, a rise of at least 50 % from baseline within the initial 7 days post-admission, or a decrease in urine output to less than 0.5 ml/kg/hour for a minimum of 6 h [27]. Patients with pre-existing conditions such as chronic kidney disease, renal replacement therapy, end-stage renal disease, malignancy, and organ transplantation were ineligible for the study. Additionally, individuals below 18 years or above 80 years of age, as well as those lacking consent, were excluded. The exclusion of patients in the higher age bracket was based on known disparities in frailty, delirium, and hospital mortality. The information for enrolled subjected was provided in Table S2.

### 2.4 Detection of blood urea nitrogen (BUN), serum creatinine (SCr) and cystatin C

To assess renal function, we measured the levels of BUN and Scr by using commercial kits obtained from Jiancheng Bioengineering Institute (Nanjing, China), following the manufacturer's protocols [28,29]. Serum levels of cystatin C were analyzed using a commercial mouse cystatin C kit obtained from Elabscience (Wuhan, China). Subsequently, the optical density for serum cystatin C measurement was determined using a microplate reader (SYNERGY H4, BioTek, VT, USA) at an absorbance of 450 nm.

### 2.5 Histological analysis

The paraffin-embedded renal or colonic tissues were cut into 5-μm thickness sections, followed by deparaffinized and rehydrated in xylene and gradient alcohol. Subsequently, tissue sections were subjected to hematoxylin eosin (HE) staining, the images were captured using a microscope (Olympus BX51) and subjected to blind analysis by a pathologist. Tubular damage severity was assessed using a semi-quantitative scoring system, considering parameters such as tubular dilation, cast formation, tubular atrophy, brush border loss, and the proportion of tubules exhibiting epithelial necrosis in the external medulla region. The average histological score for each renal sample was then calculated [29,30]. The colonic samples underwent assessment for alterations in mucosal architecture, cellular infiltration, inflammation, goblet cell depletion, surface epithelial cell hyperplasia, and indications of epithelial regeneration as previously described [31,32].

### 2.6 Quantitative real time-PCR

RNA from tissues or cells was extracted using TRIzol (Cowin, Nanjing, China), following the manufacturer's guidelines. Then, 1 μg of RNA was reverse-transcribed to cDNA using Hifair® AdvanceFast 1st Strand cDNA Synthesis Kit (YEAEN, Shanghai, China). Afterwards, we used the cDNA and Mixture and primers to prepare the qPCR reaction mix using Hieff® qPCR SYBR Green Master Mix (YEAEN, Shanghai, China) according to the suggested protocol under LightCycler 480 II system (Roche). Gene expression was normalized against β-actin and quantified with the 2^−ΔΔCt^ method. This approach accurately quantifies gene expression changes. The primers used in the present study were listed in Table S3.

### 2.7 Western blot analysis

For Western blot analysis, the collected tissues and cells were lysed using RIPA lysis buffer (Beyotime, Shanghai, China) and then centrifuged to isolate the supernatant containing total protein. The protein samples were separated on an 8–12 % SDS-PAGE gel and transferred onto polyvinylidene difluoride (PVDF) membranes. Following blocking with 5 % skimmed milk in Tris-buffered saline with Tween 20 (TBST) buffer, the membranes were incubated with primary antibodies against NGAL (26991-1-AP, Proteintech), KIM-1 (orb350868, Biorbyt), Occludin (ab216327, Abcam), ZO-1 (ab307799, Abcam), Bax (2772, Cell Signaling Technology), Cleaved-caspase3 (9661, Cell Signaling Technology), Bcl-2 (3498, Cell Signaling Technology), p65-NFκB (8242, Cell Signaling Technology), Lamin B1 (13435, Cell Signaling Technology), NLRP3 (15101, Cell Signaling Technology), ASC (10500-1-AP, Proteintech), IL-1β (ab283818, Abcam), IL-18 (10663-1-AP, Proteintech) and β-actin (66009-1-Ig, Proteintech). Subsequently, the membranes were probed with horseradish peroxidase (HRP)-conjugated secondary antibodies, visualized using enhanced chemiluminescence (ECL) reagents, and captured using the Tanon-5200 Multi chemiluminescent imaging system (Tanon, Shanghai, China).

### 2.8 Enzyme linked immunosorbent assay (ELISA)

Serum levels of TNF-α (EK0527) and IL-6 (EK0411) were measured using the commercial ELISA kits obtained from BOSTER Biological Technology (Wuhan, China) in compliance with the manufacturer's instructions [28]. Commercial kits for mouse LPS (KT37561, MSKBIO) were used for the measurement of serum LPS levels in mice.

### 2.9 DHE staining and TUNEL analysis

Intracellular reactive oxygen species (ROS) levels in renal tissues and HK-2 cells were measured using the DHE probe. Samples were treated with DHE (10 μM) for 30 min in a light-protected humidified chamber. Fluorescence signals were captured using the fluorescence microscope (Nikon, Japan) and quantified using Image-Pro Plus software (Version 6.0, Media Cybernetics, Bethesda, MD, USA) with consistent parameters. Renal cell death was assessed using the TdT-mediated dUTP Nick-End Labeling (TUNEL) in situ cell death detection kit, following the manufacturer's instructions. Click-iT Plus TUNEL Alexa Fluor™ 594 kit (Thermo) was used in cellular and animal experiments. Apoptotic cells displaying red fluorescence were detected using fluorescence microscopy (Nikon, Japan).

### 2.10 Antibiotic treatment and FMT in mice

For *in vivo* antibiotic administratio, the mice received combined antibiotics (ABX) containing neomycin (100 μg/mL), streptomycin (50 μg/mL), penicillin (100 U/mL), vancomycin (50 μg/mL), metronidazole (100 μg/mL), bacitracin (1 mg/mL), ciprofloxacin (125 μg/mL), gentamicin (170 μg/mL), and chloramphenicol (10 μg/mL) dissolved in sterile water for 7 days as previously described [33]. Following the ABX treatment, the mice underwent CR for 4 weeks and then subjected to renal IRI surgery. For microbiota transplantation, fecal samples (100 mg per mouse) were collected under sterile conditions and resuspended in pre-cooled PBS. The suspension was then centrifuged at 1000 rpm for 5 min at 4 °C, and this step was repeated twice to obtain the supernatant. Glycerin (20 %) was added before storing the samples at −80 °C. The control and CR groups were the donor mice. After one week of ABX administration, the mice were orally inoculated with the fecal suspension (200 μL per mouse) at one-day intervals for 4 weeks. The renal function and histopathological changes were assessed after renal IRI surgery.

### 2.11 Gut microbial analysis

Upon collection, the caecal content samples were promptly snap-frozen and stored at −80 °C. Bacterial DNA extraction from the caecal contents was performed using the DNeasy PowerSoil kit (Qiagen, Hilden, Germany) according to the manufacturer's protocol. Subsequently, DNA concentration and integrity were assessed using a NanoDrop 2000 spectrophotometer (Thermo Fisher Scientific, Waltham, MA, USA) and agarose gel electrophoresis, respectively. PCR amplification of the V3–V4 hypervariable regions of the bacterial 16S rRNA gene was conducted in a 25 μl reaction utilizing universal primer pairs (343F: 5′-TACGGRAGGCAGCAG-3′; 798R: 5′-AGGGTATCTAATCCT-3′). The reverse primer incorporated a sample barcode, and both primers were linked with an Illumina sequencing adapter. The Amplicon quality was visualized using gel electrophoresis. The PCR products were purified with Agencourt AMPure XP beads (Beckman Coulter Co., USA) and quantified using Qubit dsDNA assay kit. The concentrations were then adjusted for sequencing. Sequencing was performed on an Illumina NovaSeq6000 with two paired-end read cycles of 250 bases each. Representative reads for each Amplicon Sequence Variant (ASV) were selected using the QIIME 2 package. These representative reads were then annotated and compared against the Silva database Version 138 (or Unite) (16S/18S/ITS rRNA) using the q2-feature-classifier with default parameters. Microbial diversity in caecal content samples was assessed using alpha diversity measures, including the Chao1 index and Shannon index. Unweighted UniFrac Principal Coordinates Analysis (PCoA) and phylogenetic tree construction were performed based on the Unifrac distance matrix generated by the QIIME software. Sequence analysis was carried out using UPARSE software, employing both the UPARSE-OTUref and UPARSE-OTU algorithms. Sequences exhibiting over 97 % similarity were grouped into the same operational taxonomic unit (OTU). Taxonomic information for each sequence was annotated using the Ribosomal Database Project (RDP) classifier. Alpha diversity metrics including Observed species, Chao1, and Shannon indices were calculated, while beta diversity was assessed through Principal Coordinates Analysis (PCoA) at the OTU level. Taxonomy analysis and quantitative biomarker analysis among different groups were performed using Linear Discriminant Analysis Effect Size (LEfSe) on the MicrobiomeAnalyst platform. The 16S rRNA gene amplicon sequencing analysis was conducted by OE Biotech Co., Ltd. (Shanghai, China). The 16S rRNA gene sequencing data have been deposited in the NCBI Sequence Read Archive (SRA) database under accession code PRJNA1090669.

### 2.12 Fecal DNA extraction and the quantitation of *P. goldsteinii* abundance

Fecal DNA extraction was carried out using the SPINeasy DNA Kit for Feces (MP Biomedicals, Santa Ana, California, USA) following the manufacturer's instructions, and the concentration was determined using a DS-11 FX spectrophotometer (DeNovix, USA). qPCR assays were conducted using the Hieff qPCR SYBR Green Master Mix (Yeasen Biotech Co. Ltd. Shanghai, China) with primers targeting the genes encoding 16S rRNA from *P. goldsteinii* (forward primer: 5′-ATTCGGCATCCTCACTTC-3’; reverse primer: 5′-ATCGTCCACGGTGTCCAG-3′) and all bacteria (forward primer: 5′-TGSTGCAYGGYTGTCGTCA-3’; reverse primer: 5′-ACGTCRTCCMCACCTTCCTC-3′) [34]. The qRT-PCR was carried out under the LightCycler 480 II system (Roche).

### 2.13 *P. goldsteinii* cultivation and preparation

*P. goldsteinii* was procured from the American Type Culture Collection (ATCC, USA) and cultivated at 37 °C within a Whitley DG250 anaerobic chamber (Don Whitley, UK) under a mixed anaerobic gas environment (5 % carbon dioxide, 5 % hydrogen, 90 % nitrogen). After cultivation, *P. goldsteinii* was harvested by centrifugation and suspended in sterile saline solution. Mice received daily oral gavage of 10^8^ colony-forming units (CFU) of live *P. goldsteinii*. Heat-killed or pasteurized *P. goldsteinii* were prepared by subjecting the bacteria to heating at 100 °C for 15 min. Mice were treated daily with either heat-killed or live *P. goldsteinii* via oral gavage for 4 weeks [17,35].

### 2.14 Metabolome analysis

Serum samples stored at −80 °C were thawed at room temperature, and 50 μL of samples and 200 μL of protein precipitant methanol acetonitrile (V: V = 2:1, containing L-2-chlorophenylalanine, 2 μG/mL) were added to 1.5 mL Eppendorf tube under vortex oscillation for 1 min. The mixture underwent ultrasound extraction in ice water bath for 10 min and stored at −40 °C for 30 min. The extract was centrifuged at 4 °C (13,000 rpm) for 10 min, and the supernatants of 150 μL were aspirated and filtered using 0.22 μm microfilter sand and then transferred to LC vials and stored at −80 °C until LC-MS analysis was performed. Quality control samples (QC) are prepared by mixing the extraction solutions of all samples in equal volumes. The analytical instrument used in this experiment was a liquid chromatography-mass spectrometry system composed of ACQUITY UPLC I-Class plus ultra-high performance liquid chromatography tandem QE high-resolution mass spectrometer. Chromatographic conditions were performed as follows: chromatographic column: ACQUITY UPLC HSS T3 (100 mm × 2.1 mm, 1.8 μm); column temperature: 45 °C; Mobile phase: A-water (containing 0.1 % formic acid), B-acetonitrile; Flow rate: 0.35 mL/min; Injection volume: 5 μL. The sample mass spectrometry signals were collected using both positive and negative ion scanning modes. The original LC MS data were processed by software Progenesis QIV2.3 (Nonlinear, Dynamics, Newcastle, UK) for baseline filtering, peak identification, integral, retention time correction, peak alignment, and normalization. Main parameters of 5 ppm precursor tolerance, 10 ppm product tolerance, and 5 % production threshold were applied. Compound identification was based on precise mass-to charge ratio (M/z), secondary fragments, and isotopic distribution using The Human Metabolome Database (HMDB), Lipidmaps (V2.3), Metlin, EMDB, PMDB, and self-built databases to do qualitative analysis. The extracted data were then further processed by removing any peaks with a missing value (ion intensity = 0) in more than 50 % in groups, by replacing zero value by half of the minimum value, and by screening according to the qualitative results of the compound. Metabolites displaying statistically significant alterations (p < 0.05) were selected and identified based on the KEGG database. Statistical analyses were performed using MetaboAnalyst 4.0. For quantification of Dodecafluorpentan, UPLC separation was performed using the ExionLC AD system from AB SCIEX, coupled with a Waters ACQUITY UPLC C18 column. The oven temperature was maintained at 40 °C, and a sample injection volume of 2 μL was utilized. Metabolites were eluted from the column at a flow rate of 0.35 mL/min, employing mobile phases consisting of 0.1 % acetic acid in water (phase A) and 0.1 % acetic acid in acetonitrile (phase B). A gradient elution program was applied as follows: 0–11 min: linear gradient from 5 % to 90 % B; 11–12 min: 90 % B; 12–12.1 min: linear gradient from 90 % to 5 % B; 12.1–14 min: 5 % B. Metabolic extracts from each sample were analyzed using a triple quadrupole-linear ion trap mass spectrometer (QTRAP 6500, AB SCIEX) in both positive and negative ionization modes. The electrospray ionization (ESI) source conditions were set as follows: ion spray voltage (IS) 5500 V (positive mode), −4500 V (negative mode); ion source gas I (GSI), gas II (GASII), and curtain gas (CUR) were maintained at 50, 50, and 25 psi, respectively. Detection of each ion pair was conducted based on optimized voltage and collision energy settings.

### 2.15 The potential agents that promoted the growth of *P. goldsteinii*

*In vitro* screening of growth modulators for *P. goldsteinii* was conducted by seeding the cultured bacteria in 96-well plates at an initial OD600 value of approximately 0.2. These cultures were then treated with 78 sourced flavonoid glycosides, each at a final concentration of 10 μM. Subsequently, the OD600 values were measured at various time points using a microplate reader to assess the absorbance. Following preliminary screening, the top-five compounds were subjected to further investigation at time intervals of 0, 6, 12, 24, 36, 48, and 72 h to observe their effects on the growth of *P. goldsteinii*.

### 2.16 Immunofluorescence

Tissue sections of 5-μm thickness were deparaffinized and rehydrated. Subsequently, the sections were blocked with 5 % Bovine Serum Albumin (BSA) for 1 h, and permeabilized with 1 % Triton X-100 for 15 min. The samples were incubated overnight with primary antibodies against ZO-1 (21773-1-AP, 1:500, Proteintech) overnight at 4 °C. On the next day, the sections were incubated with Goat Anti-Rabbit IgG H&L (Alexa Fluor® 488) for 1 h at room temperature and stained with DAPI. In addition, the immunofluorescence staining of p65 NF-κB was conducted using the primary antibody against of p65 NF-κB (80979-1-RR), and the Goat Anti-Rabbit IgG H&L (Alexa Fluor® 488, ab150077, Abcam) and Goat Anti-Rabbit IgG H&L (Alexa Fluor® 594, ab150080, Abcam) were used for cells and renal sections, respectively. The images were conducted using an Axio Imager Z2 microscope, and the analysis was carried out with ImageJ software.

### 2.17 Immunohistochemistry

After the procedure of dewaxing, hydration, endogenous peroxidase inactivation and blocking with sheep serum, the colonic sections were probed with the required primary antibodies, including anti-occludin antibody (27260-1-AP, Proteintech) overnight at 4 °C. The sections were then incubated with a biotinylated streptavidin-peroxidase complex for 1 h. Subsequently, the slides were stained using DAB solution followed by hematoxylin staining. The stained cells in each section were captured under a photomicroscope (Olympus, Tokyo, Japan). The positive area of occludin was measured by ImageJ software.

### 2.18 Cell culture

HK-2 cells were cultured in DMEM/F12 medium with 10 % FBS and 1 × penicillin-streptomycin. The culture medium was changed for every 2 days, and the cells would be passaged before reaching 80–90 % confluence. They were maintained in an incubator set at 37 °C with 5 % CO_2_. For mimicking ischemic conditions *in vitro*, an oxygen-glucose deprivation/reoxygenation (OGD/R) model had been applied in HK2 cells. The cells were placed in a 1 % nitrogen (N_2_) cell culture incubator to induce hypoxia for 12 h, followed by reoxygenation in a 5 % CO_2_ incubator for 2 h.

### 2.19 Cell counting kit-8 (CCK-8) assay

The CCK-8 assay was utilized to assess the impact of Dodecafluorpentan on HK-2 cell viability and determine the optimal concentration of Dodecafluorpentan. Initially, 1 × 10^4^ cells in 100 μL of medium were seeded into each well of a 96-well plate and allowed to incubate overnight. Following this, the cells were exposed to various concentrations of Dodecafluorpentan (ranging from 0 to 200 μM) for 48 h. After treatment, the medium was replaced with 100 μL of medium containing 10 % CCK-8 and incubated at 37 °C in the absence of light for 1 h. The absorbance was then measured at 450 nm using a multifunctional enzyme labeler (Biotek Synergy H4, USA).

### 2.20 Lactate dehydrogenase (LDH) release

The LDH assay was conducted by collecting the supernatant from each group of HK-2 cells. The supernatants were then sequentially transferred to a 96-well plate following the instructions provided with the LDH kit from Nanjing Jiancheng Bioengineering Institute (Nanjing, China). After incubation, absorbance readings were taken at a wavelength of 450 nm using a microplate reader. LDH activity for each group of HK-2 cells was subsequently calculated.

### 2.21 Annexin V– PI staining

To evaluate apoptosis in HK-2 cells, we tracked the presence of phosphatidylserine on their outer membranes using Annexin V. Initially, we treated cells with trypsin, then washed them meticulously with PBS. Subsequently, the cells were incubated in a special binding buffer that included both Annexin V and propidium iodide, employing the Annexin V-FITC Apoptosis Detection Kit (Beyotime Biotechnology) for the staining process. Flow cytometry analysis was performed using Accuri C6 Plus.

### 2.22 RNA sequencing

Transcriptome sequencing and analysis were performed by OE Biotech Co., Ltd. (Shanghai, China), as previously described [36,37]. Raw RNA sequencing data have been deposited in the Sequencing Read Archive (SRA) under accession number PRJNA1091143.

### 2.23 Statistical analysis

The experimental results, reported as means ± SEM, underwent analysis employing various statistical methods, including the Wilcoxon signed-rank test, Student's t-test, and one-way or two-way analysis of variance (ANOVA), to compare numerical variables. Normality of the data was assessed using the Shapiro-Wilk test and quantile-quantile plots. Statistical comparisons between two groups were conducted using Student's t-test, while comparisons among multiple groups utilized ANOVA/Bonferroni-test. Proportions of categorical variables were evaluated using either Chi-square or Fisher's exact tests. Statistical analyses were performed using SPSS (version 19.0) or GraphPad Prism (version 7.0) software. A significance level of *p* less than 0.05 was considered statistically significant.

## 3 Results

### 3.1 CR protects the kidneys from damage induced by renal IRI

**Fig. 1:**
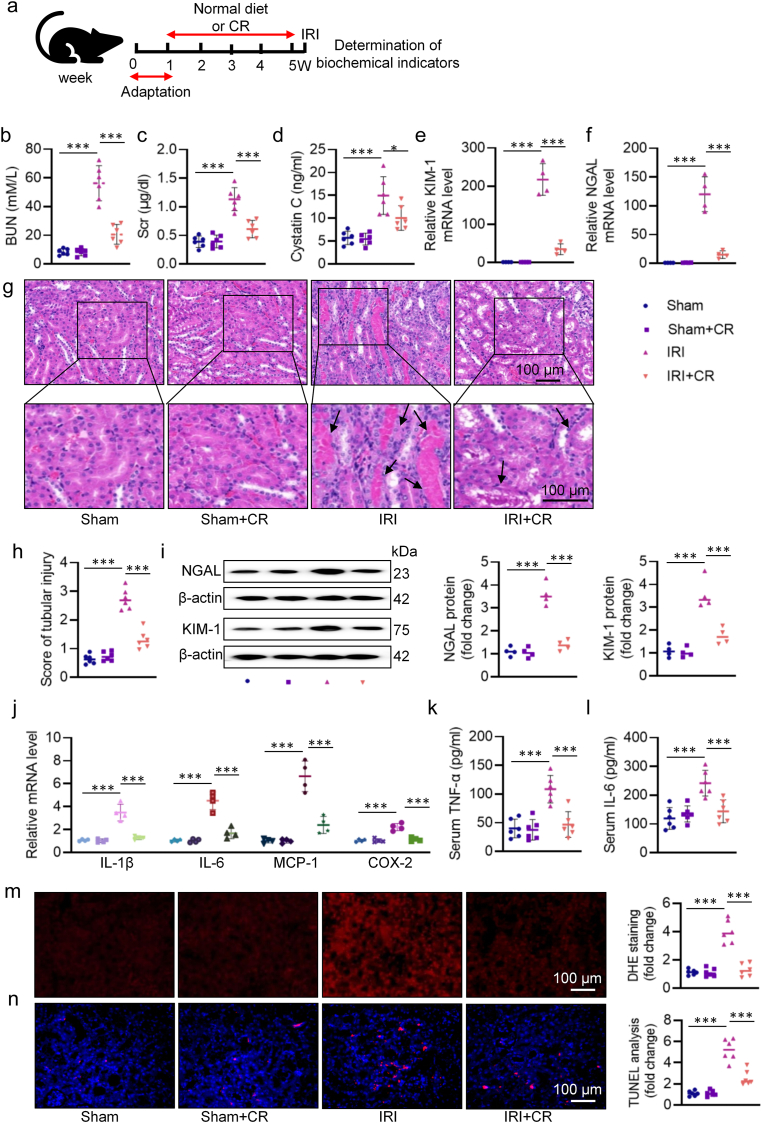
CR treatment attenuates IRI-induced kidney dysfunction and intestinal injury. **a** Schematic diagram of the experimental design. **b** Serum BUN levels. **c** Scr levels. **d** Serum cystatin C levels. **e, f** The mRNA levels of KIM-1 and NGAL. **g** Representative HE images of kidney. Arrows indicate injured tubules. **h** Analysis of tubular injury score in mice. **i** Representative western blots and quantitative analysis of NGAL and KIM-1. **j** The mRNA levels of IL-1β, IL-6, MCP-1, and COX-2. **k, l** The levels of serum TNF-α and IL-6. **m** Representative images and quantitative analysis of DHE staining (Scale bar = 100 μm). **n** Representative images and quantitative analysis of TUNEL staining (Scale bar = 100 μm). ∗P < 0.05, ∗∗P < 0.01, ∗∗∗P < 0.001 versus the indicated group.

Bilateral renal ischemia was induced by clamping both renal pedicles for 30 min, followed by clamp removal to reinitiate blood flow for 24 h. To evaluate the potential impact of CR, male C57BL/6J mice were assigned to a normal diet or CR regimen (70 % of the averaged daily intake) for 28 days (Fig. 1a). After that, the mice underwent renal IRI or sham surgery. Compared to mice fed *ad libitum*, those subjected to CR experienced approximately 15 % body weight loss before surgery (Table S4). However, CR intervention prevented IRI-induced increases in BUN, Scr, and cystatin C (Fig. 1b–d), along with decreased mRNA levels of kidney injury molecule 1 (KIM-1) and neutrophil gelatinase associated lipocalin (NGAL) in AKI mice (Fig. 1e–f). HE staining of renal sections indicated that IRI induced injury to kidneys, characterized by tubular cell necrosis, renal tubule dilation, protein cast formation in renal tubules, effects that were prevented by CR (Fig. 1g–h). Similar to mRNA changes, the protein expression of NGAL and KIM-1 was suppressed by CR in AKI kidneys (Fig. 1i). As shown in Fig. 1j–l, inflammatory response was strikingly induced in response to renal IRI, as shown by upregulated mRNA levels of IL-1β, IL-6, MCP-1 and COX-2 in the kidneys, as well as increased serum TNF-α and IL-6 levels. Nevertheless, these abnormalities were corrected by CR (Fig. 1j–l). DHE and TUNEL staining results showed higher oxidative damage and cell apoptosis in kidneys of AKI mice, which were markedly ameliorated by CR (Fig. 1m–n). A separate body of research implicates that intestinal barrier dysfunction has been taken as a key priming stage for renal injury after IRI [1]. Histopathological sections of the intestinal tract of AKI mice showed significant surface loss of epithelial cells and expansion of capillaries, together with increased endotoxin levels (Figs. S1a–b). Conversely, CR treatment reversed these changes (Figs. S1a–b). In keeping with this, CR conserved intestinal permeability in mice with renal IRI, as reflected by increased protein and mRNA expression levels of occludin and ZO-1, two tight junction proteins involved in maintaining intestinal barrier integrity (Figs. S1c–d). Immunohistochemistry and immunofluorescence further confirmed that CR treatment restored the protein expression of occludin and ZO-1 (Figs. S1e–h). TEM analysis of colonic tissues was carried out to assess the structure of the intestinal barrier. TEM results revealed that the colonic epithelial cells were damaged in mice with renal IRI, as indicated by disorganized and sparse microvilli, a reduced amount of or discontinuity of intercellular electron-dense material, and widened intercellular spaces. However, these changes were significantly reversed by CR intervention (Fig. S2a). These results indicate that CR preserved renal function and histology, and improved colon permeability in mice following renal IRI.

### 3.2 Antibiotic treatment weakened the protective effects of CR in renal dysfunction post-IRI

**Fig. 2:**
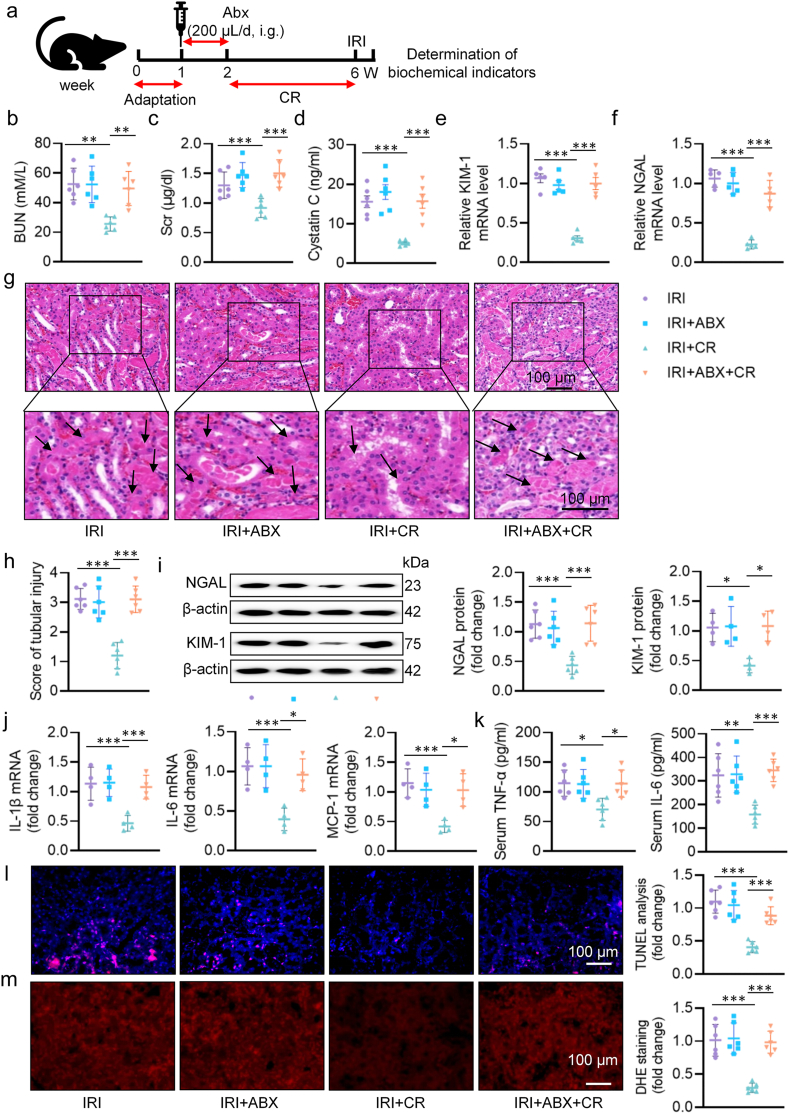
The effect of gut microbiota deletion on IRI-induced renal damage. **a** Schematic diagram of the experimental design. **b** Serum BUN levels. **c** Scr levels. **d** Serum cystatin C levels. **e, f** The mRNA levels of KIM-1 and NGAL. **g** Representative HE images of kidney. Arrows indicate injured tubules. **h** Analysis of tubular injury score in mice. **i** Representative western blots and quantitative analysis of NGAL and KIM-1. **j** The mRNA levels of IL-1β, IL-6, and MCP-1. **k** The levels of serum TNF-α and IL-6. **l** Representative images and quantitative analysis of TUNEL staining (Scale bar = 100 μm). **m** Representative images and quantitative analysis of DHE staining (Scale bar = 100 μm). ∗P < 0.05, ∗∗P < 0.01, ∗∗∗P < 0.001 versus the indicated group.

To explore whether the gut microbiota is primarily responsible for the beneficial actions of CR on renal dysfunction post-IRI, we administered oral antibiotics to male mice for 7 days to deplete the gut microbiota and started CR treatment for 4 weeks (Fig. 2a), ABX treatment did not affect the effect of CR on body loss in mice (Table S5). Intriguingly, ABX supplementation disrupted renal function of CR-treated mice, as evidenced by the loss of benefits of CR in renal IRI mice, including the levels of BUN, Scr and cystatin C (Fig. 2b–d), the mRNA levels of KIM-1 and NGAL (Fig. 2e–f), renal histology (Fig. 2g–h), the protein expression of NGAL and KIM-1 (Fig. 2i), inflammation (Fig. 2j–k), oxidative stress (Fig. 2l), and renal cell apoptosis (Fig. 2m). In the meantime, the benefits of CR in intestinal permeability were largely eliminated by ABX treatment, as indicated by HE staining of colonic sections (Figs. S3a and d), immunohistochemistry of occludin (Figs. S3b and e), immunofluorescence staining (Figs. S3c and f), serum LPS levels (Fig. S3g), immunoblotting and RT-PCR analysis of occludin and ZO-1 (Figs. S3h and i), as well as TEM assay (Fig. S2a). Thus, these observations confirmed that the gut microbiota may be responsible for the protective effects of CR in renal dysfunction post-IRI.

### 3.3 Fecal microbial transplantation (FMT) from CR-treated mice provides renoprotection

**Fig. 3:**
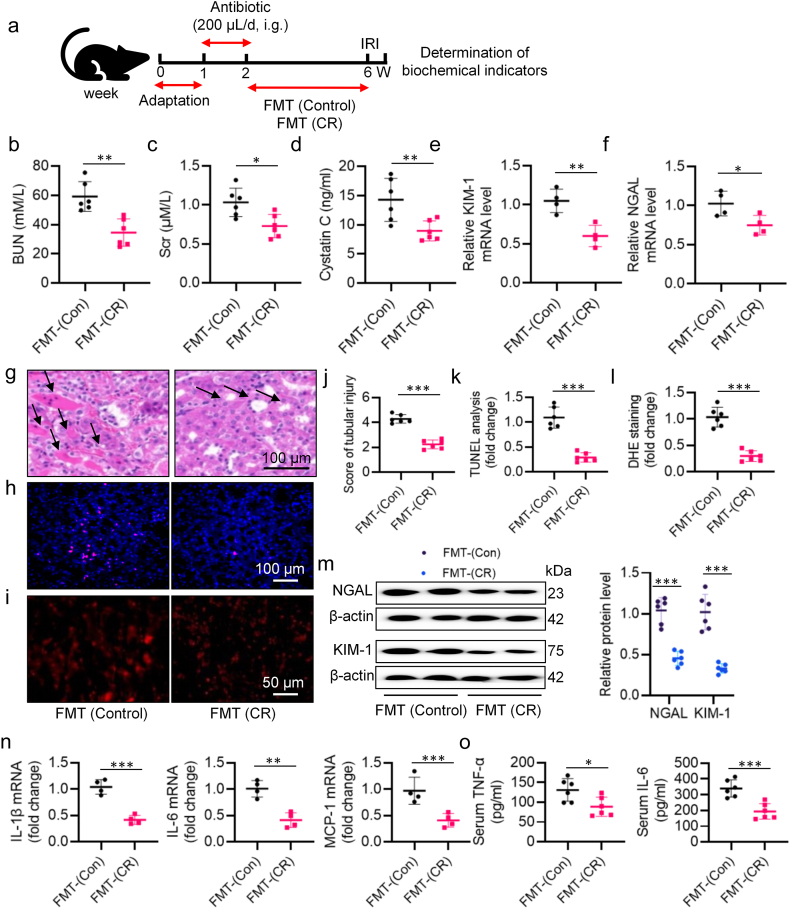
FMT recovers the protection of CR. **a** The schedule of FMT after ABX. **b** Serum BUN levels. **c** Scr levels. **d** Serum cystatin C levels. **e, f** The mRNA levels of KIM-1 and NGAL. **g** Representative HE images of kidney. Arrows indicate injured tubules. **j** Analysis of tubular injury score in mice. Representative images **h** and quantitative analysis **k** of TUNEL staining (Scale bar = 100 μm). Representative images (**i)** and quantitative analysis (I**)** of DHE staining (Scale bar = 50 μm). **m** Representative western blots and quantitative analysis of NGAL and KIM-1. **n** The mRNA levels of IL-1β, IL-6, and MCP-1. **o** The levels of serum TNF-α and IL-6. ∗P < 0.05, ∗∗P < 0.01, ∗∗∗P < 0.001 versus the indicated group.

To establish the causal relationship between the gut microbiota and the attenuating effect of CR on renal IRI, we performed FMT following oral broad-spectrum antibiotics for 7 days. Herein, we assessed renal injury through transplanting the gut microbiota using fecal samples from CR-treated mice (Fig. 3a). The body weight was not changed by FMT treatment (Table S6). We found that mice undergoing feces transplantation from CR-treated mice displayed lower BUN, Scr, and cystatin C levels upon exposure to renal IRI surgery (Fig. 3b–d). Compared with mice of the FMT from control group, the mRNA levels of KIM-1 and NGAL were apparently downregulated by FMT from CR group (Fig. 3e–f). Additionally, renal morphology was improved in mice transplanted with fecal microbiota from the CR group (Fig. 3g, j). Western blotting further revealed that protein level of KIM-1 and NGAL was decreased by FMT from the CR group (Fig. 3m). As expected, mice transplanting fecal microbiota from CR-treated mice had less renal cell apoptosis (Fig. 3h, k) and oxidative injury (Fig. 3i, l) and inflammatory response (Fig. 3n–o). Next, we detected the intestinal barrier function of the colon. HE staining demonstrated the FMT from CR-treated mice rescued the intestinal damage induced after IR-induced injury, compared with FMT from control mice (Figs. S4a and d). We observed that mice receiving microbiota transplants from CR-treated mice exhibited preserved barrier permeability, as evidenced by increased expression of ZO-1 and occluding (Fig. S4b-c, e-f). In keeping with the immunostaining results, the protein and mRNA levels of ZO-1 and occludin tended to be higher in colon tissues of mice receiving faecal microbiota from CR-treated mice (Figs. S4g–i). Therefore, CR treatment alleviated IRI-induced kidney dysfunction and intestinal injury.

### 3.4 *Parabacteroides goldsteinii* (*P. goldsteinii*) contributes to CR-induced renoprotection

**Fig. 4:**
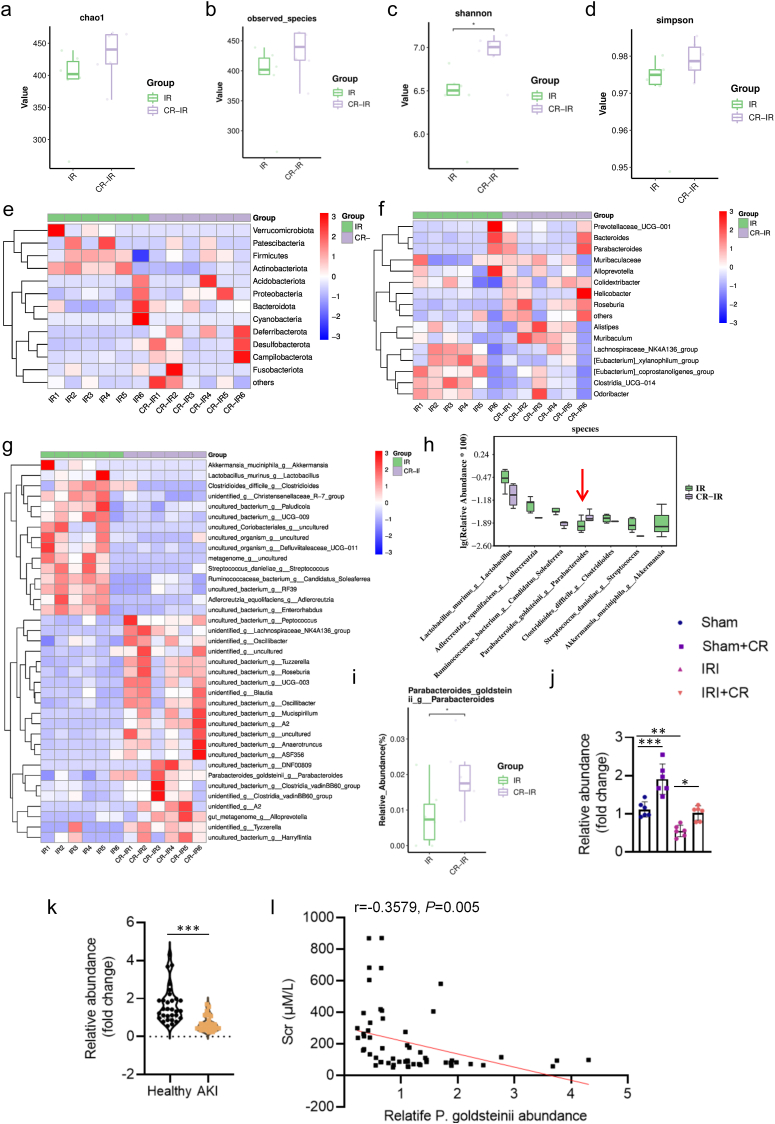
CR treatment alters the richness and community diversity of gut microbiome in mice induced by IRI. **a-d** Alpha diversity of the intestinal flora between IR group and CR-treated IRI group. **e** Relative abundances of intestinal flora at phylum level. **f** Relative abundances of intestinal flora at family level. **g-i** Relative abundances of intestinal flora at genus level. **j** Relative abundance of *P. goldsteinii* by qPCR in mice. **k** Relative abundance of *P. goldsteinii* by qPCR in patients. **l** The negative association of serum Scr level with *P. goldsteinii* contents by a Pearson correlation analysis. ∗P < 0.05, ∗∗P < 0.01, ∗∗∗P < 0.001 versus the indicated group.

To discern the specific gut microbial components responsible for the renal benefits of CR in the setting of renal IRI, the fecal samples were collected from CR-treated mice and subjected to 16S rRNA sequencing. Our analysis revealed that the diversity and richness of the gut microbial community were slightly elevated in IRI mice undergoing CR treatment, as evidenced by metrics such as Chao 1, Observed species, Shannon, and Simpson indices (Fig. 4a–d, S5a). At the phylum level, the gut microbiome was perturbed in mice following renal IRI procedure, characterized by a decrease in *Bacteroidetes* and an increase in *Firmicutes*, which was ameliorated by CR treatment (Fig. 4e). Furthermore, compared to mice in the IRI group, those in the CR group exhibited notably higher levels of *Bacteroides*, *Parabacteroides*, and *Muribaculaceae* (Fig. 4f, S5b). Subsequently, a comprehensive examination of the intestinal flora at the genus level between IR mice and those subjected to IR with CR treatment revealed significant downregulation of *Lactobacillus_murinus*, *Adlercreutzia_equolifaciens*, *Ruminococcaceae_bacterium*, *Clostridioides_difficile*, *Streptococcus_danieliae*, and *Akkermansia_muciniphila*, while *P. goldsteinii* exhibited marked upregulation in CR-treated mice (Fig. 4g–i, S6). Notably, the relative abundance of *P. goldsteinii* was substantially diminished in AKI mice induced by renal IRI, but was restored by CR treatment (Fig. 4j). Additionally, the abundance of *P. goldsteinii* was found to be lower in AKI patients compared to the healthy individuals (Fig. 4k). Pearson's correlation analysis revealed a negative correlation between Scr levels and the abundance of *P. goldsteinii* (Fig. 4l). Taken together, CR treatment may mitigate renal IRI by modulating the composition of the intestinal flora, particularly by enhancing the presence of *P. goldsteinii*.

### 3.5 Effects of *P. goldsteinii* on renal IRI in mice

**Fig. 5:**
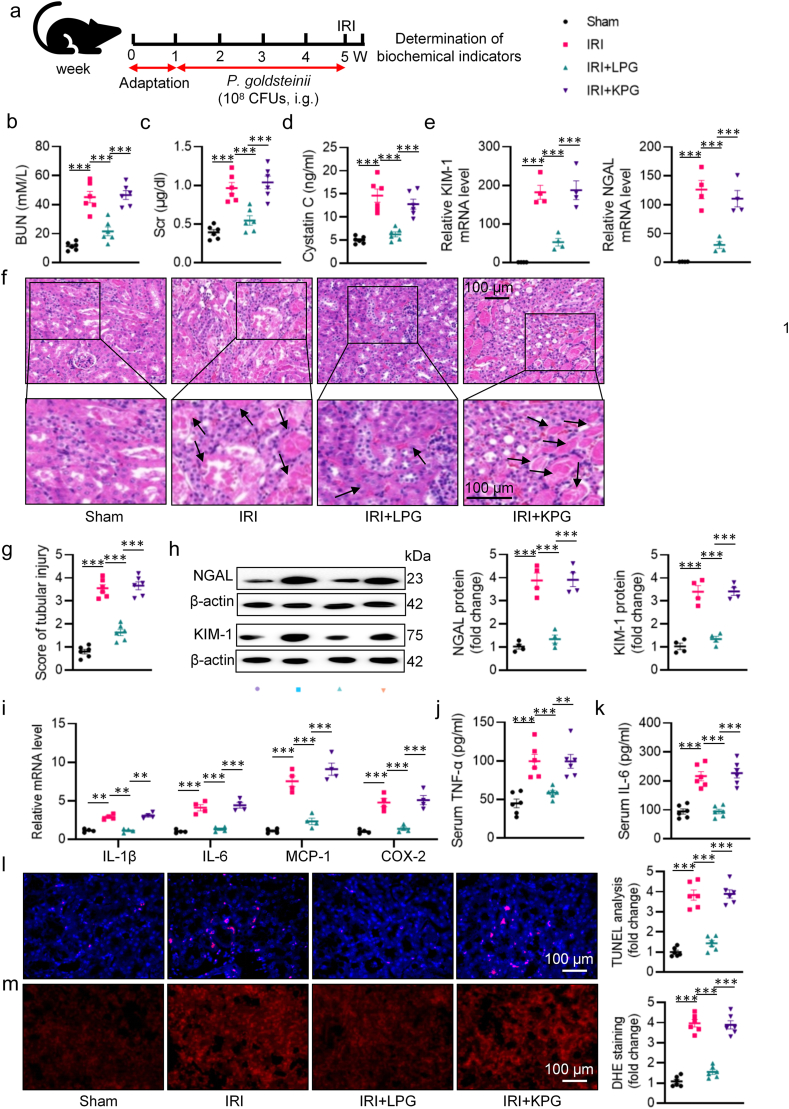
The effect of *P. goldsteinii* on IRI-induced renal injury. **a** The schedule of supplementation of *P. goldsteinii* after ABX. **b** Serum BUN levels. **c** Scr levels. **d** Serum cystatin C levels. **e** The mRNA levels of KIM-1 and NGAL. **f** Representative HE images of renal sections. Arrows indicate injured tubules. **g** Analysis of tubular injury score in mice. **h** Representative western blots and quantitative analysis of NGAL and KIM-1. **i** The mRNA levels of IL-1β, IL-6, MCP-1 and COX-2. **j-k** The levels of serum TNF-α and IL-6. **l** Representative images and quantitative analysis of TUNEL staining (Scale bar = 100 μm). **m** Representative images and quantitative analysis of DHE staining (Scale bar = 100 μm). ∗P < 0.05, ∗∗P < 0.01, ∗∗∗P < 0.001 versus the indicated group.

To evaluate the potential beneficial effects of *P. goldsteinii* on renal dysfunction, male C57BL/6 mice received *P. goldsteinii* (10^8^ CFUs, daily) via intragastric gavage for 4 weeks prior to undergoing renal IRI procedure (Fig. 5a). Oral administration of *P. goldsteinii* had no impact on body weight (Table S7). Our findings revealed that live *P. goldsteinii* (LPG) significantly attenuated the elevated levels of BUN, Scr, and cystatin C induced by renal IRI, whereas killed *P. goldsteinii* (KPG) did not produce similar effects (Fig. 5b–d). Additionally, results obtained from qPCR and western blotting analyses demonstrated that LPG reduced the expression of renal tubular injury markers, such as KIM-1 and NGAL, following the IRI procedure (Fig. 5e, h). HE staining of renal sections revealed observable tubular injury occurred after AKI induction, which was reversed by LPG treatment (Fig. 5f, g). We investigated whether LPG affected IRI-induced inflammation cytokine overproduction, renal cell apoptosis, and oxidative burst. Our results indicated that LPG treatment notably reduced inflammation response, renal cell apoptosis, and ROS accumulation in the context of IRI, as evidenced by decreased mRNA levels and release of inflammatory factors (Fig. 5i–k), DHE staining (Fig. 5l), and TUNEL staining (Fig. 5m). Additionally, supplementation of LPG appeared to protect the intestinal barrier from destruction, as evidenced by HE staining, immunohistochemistry and immunofluorescence analysis of occludin and ZO-1, occludin and ZO-1 protein and mRNA levels measured by Western blotting and RT-PCR (Fig. S7), as well as TEM assay (Fig. S2a). Importantly, all the changes induced by renal IRI mentioned above were not influenced by KPG (Fig. 5 and S7). Overall, *P. goldsteinii* demonstrated the ability to mimic renoprotection and intestinal protection under CR treatment.

### 3.6 Supplementation of dodecafluorpentan alleviates the renal dysfunction after IRI

**Fig. 6:**
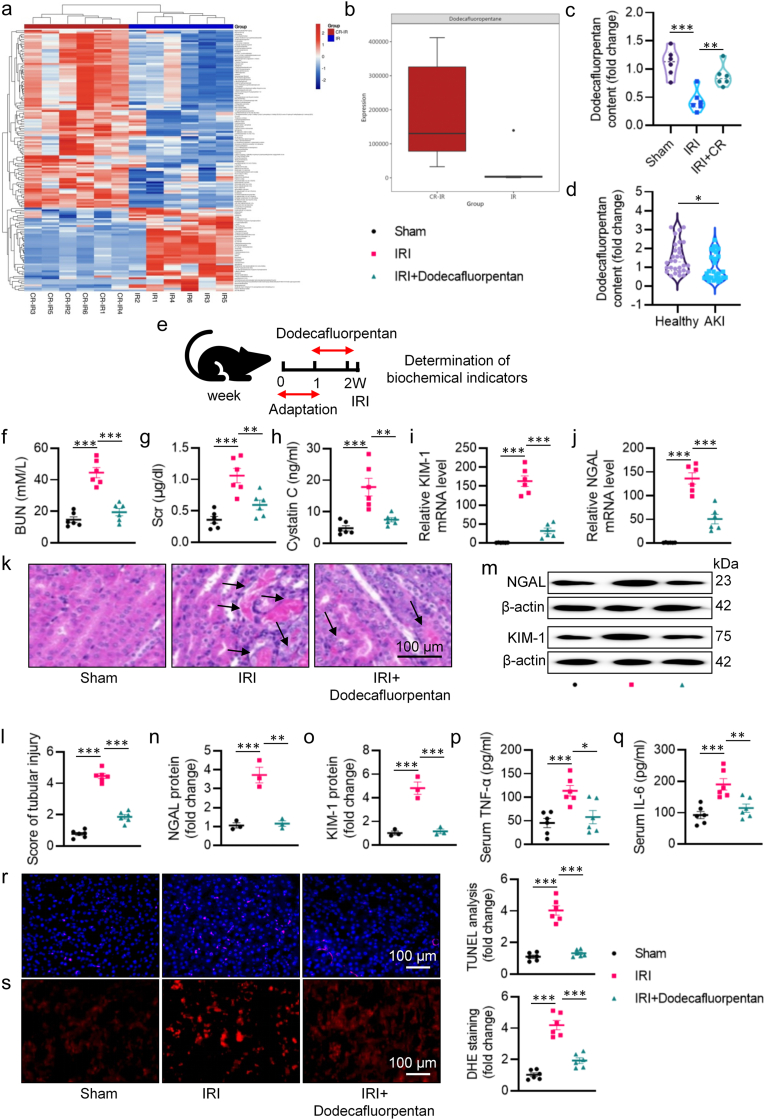
The effect of dodecafluorpentan on IRI-induced renal injury. **a** Heatmap of differentially metabolite expression between IR group and CR-treated IRI group. **b** The level of dodecafluorpentan by metabolomics. **c** The level of dodecafluorpentan in mice. **d** The level of dodecafluorpentan in AKI patients. **e** Schematic diagram of the experimental design. **f** Serum BUN levels. **g** Scr levels. **h** Serum cystatin C levels. **i, j** The mRNA levels of KIM-1 and NGAL. **k** Representative HE images of kidney. Arrows indicate injured tubules. **l** Analysis of tubular injury score in mice. **m** Representative western blots of NGAL and KIM-1. Quantitative analysis of NGAL (**n**) and KIM-1 (**o**). **p, q** The levels of serum TNF-α and IL-6. **r** Representative images and quantitative analysis of TUNEL staining. **s** Representative images and quantitative analysis of DHE staining. ∗P < 0.05, ∗∗P < 0.01, ∗∗∗P < 0.001 versus the indicated group.

To investigate whether metabolite disturbances are associated with the pathological processes of renal IRI in mice, we collected serum samples from IRI mice and CR-treated IRI mice and conducted untargeted and targeted metabolomics analysis to detect their metabolic characteristics. Overall, a total of 150 metabolites were differentially quantitated using ultraperformance liquid chromatography coupled to tandem mass spectrometry (UPLC-MS/MS) system (Fig. 6a, Table S8). These metabolites included glycerophospholipids, organofluorides, fatty acyls, peptidomimetics, prenol lipids and imidazopyrimidines (Fig. 6a–b). Our metabolomics analysis revealed that the increase in dodecafluoropentan levels was most significant in IRI mice treated with CR (Fig. 6a). Thus, we next explored the pharmacological potential of dodecafluoropentan in the context of renal IRI since metabolites may exhibit regulatory functions in the body due to their ubiquitous existence. Consistently, the content of dodecafluorpentan was decreased in the IRI group compared to the sham group, whereas it was dramatically increased in the IRI group treated with CR (Fig. 6c). Additionally, serum levels of dodecafluorpentan in AKI patients were significantly lower than those in healthy individuals (Fig. 6d). Next, we directly administered dodecafluorpentan via oral gavage for one week prior to the initiation of IRI to validate its renoprotective effects (Fig. 6e). We found that dodecafluorpentan ameliorated the pathogenesis of AKI triggered by IRI, as evidenced by reduced levels of BUN (Fig. 6f), Scr (Fig. 6g), and cystatin C (Fig. 6h), as well as the preservation of renal structure (Fig. 6k and l). Both qPCR (Fig. 6i–j) and western blotting (Fig. 6m–o) demonstrated that the protein expression of KIM-1 and NGAL induced by IRI were markedly decreased upon supplementation with dodecafluorpentan. Moreover, dodecafluorpentan significantly attenuated IRI-induced inflammation, apoptosis, and oxidative stress (Fig. 6p–s). Additionally, administration of dodecafluorpentan reversed gut leaky, restored expressions of occludin and ZO-1, and inhibited LPS release induced by renal IRI (Figs. S2a and S8). Moreover, dodecafluorpentan treatment had no influence on the body weight in mice (Table S9). These results indicate that dodecafluorpentan protects the ischemic kidney and intestinal epithelial barrier from damage. Furthermore, we explored the effect of dodecafluorpentan on HR-evoked HK-2 cell insult. Dodecafluorpentan had no effect on cell viability in HK-2 cells at different concentrations (Fig. S9a), whereas it significantly relieved HR-triggered reduction in cell viability (Fig. S9b). HR facilitated the release of LDH in HK-2 cells, and this was inhibited in the presence of dodecafluorpentan (Fig. S9c). Moreover, we found that dodecafluorpentan ameliorated HR-induced cell apoptosis and oxidative damage in HK-2 cells (Figs. S9d–k).

### 3.7 Dodecafluorpentan confers renoprotection by suppressing NFκB-mediated NOD-like receptor thermal protein domain-associated protein 3 (NLRP3) inflammasome activation

**Fig. 7:**
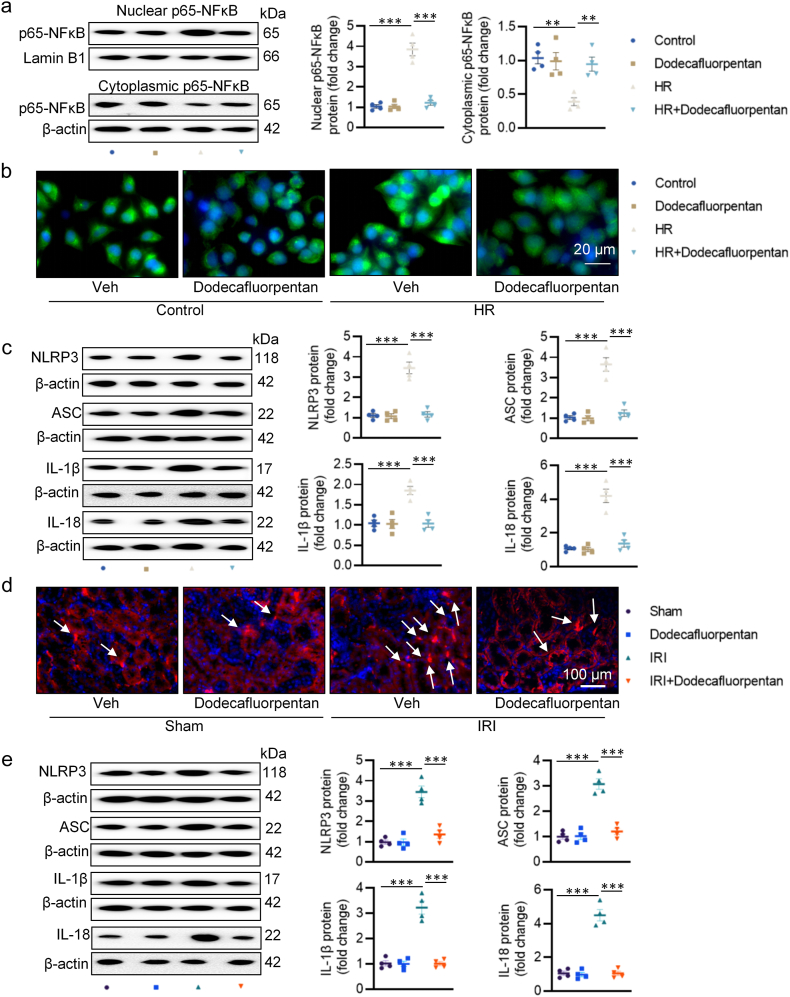
Effect of dodecafluorpentan on NFκB-induced inflammation in HR-stimulated HK-2 cells or IR-induced renal damage. **a** The protein expression of p65-NFκB in the nucleus and cytoplasm. **b** Immunofluorescence staining of p65-NFκB in HK-2 cells. Arrows indicate the p65-NFκB-positive cells. Scale bar, 20 μm. **c** Western blot analysis of NLRP3, ASC, IL-1β, and IL-18 in HK-2 cells. **d** Immunofluorescence staining of p65-NFκB in kidney. Scale bar, 100 μm. **e** Western blot analysis of NLRP3, ASC, IL-1β, and IL-18 in kidney. ∗P < 0.05, ∗∗P < 0.01, ∗∗∗P < 0.001 versus the indicated group.

To explore the potential downstream mediators of dodecafluorpentan, we conducted RNA sequencing on HK-2 cells subjected to HR stimulation with or without dodecafluorpentan treatment. Following dodecafluorpentan administration, we observed 914 upregulated genes and 1204 downregulated genes in HR-treated HK-2 cells (Fig. S10a). Protein-protein interaction (PPI) analysis highlighted NFκB1 as a core protein (Fig. S10b). Gene ontology analysis revealed significantly exacerbated inflammatory responses, particularly involving MAPK and NFκB signaling pathways, as well as the IL-18 signaling pathway (Fig. S10c). Moreover, gene set enrichment analysis indicated a clear positive correlation between dodecafluorpentan treatment and the NOD-like receptor signaling pathway (Fig. S10d). To investigate whether NFκB signaling inactivation contributed to the renoprotective effects of dodecafluorpentan, we performed western blotting and immunofluorescence for p65-NFκB. HR exposure promoted nuclear localization of p65-NFκB and inhibited its expression in the cytoplasm, effects that were prevented by dodecafluorpentan (Fig. 7a–b). Additionally, HR-induced upregulations of NLRP3, ASC, IL-1β, and IL-18 protein expressions were attenuated by dodecafluorpentan treatment (Fig. 7c). Consistent with our *in vitro* findings, dodecafluorpentan administration mitigated IRI-induced nuclear translocation of p65-NFκB and increased protein expression of NLRP3, ASC, IL-1β, and IL-18 in kidneys from AKI mice (Fig. 7d–e). These results collectively suggest that dodecafluorpentan protects against renal injury by the restraining NFκB signaling pathway and inhibiting NLRP3 inflammasome activation.

### 3.8 Screening of potential candidates to promote the growth of *P. goldsteinii*

**Fig. 8:**
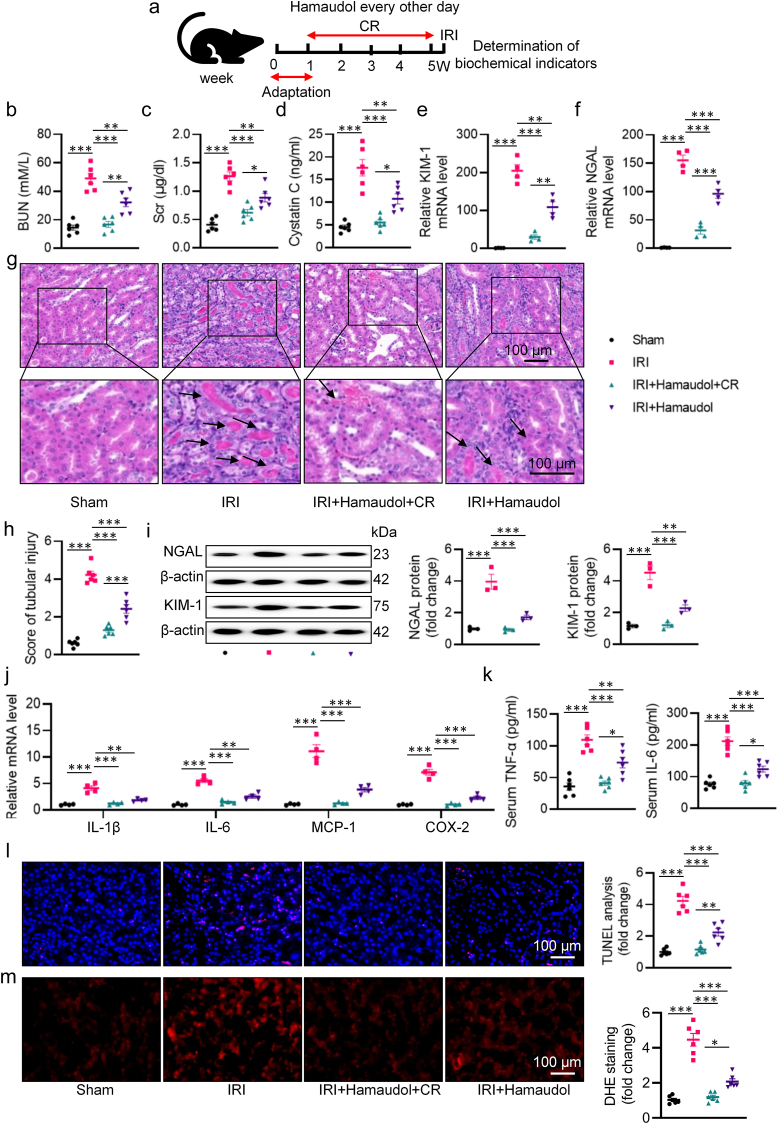
The effect of Hamaudol on IRI-induced renal injury. **a** Schematic diagram of the experimental design. **b** Serum BUN levels. **c** Scr levels. **d** Serum cystatin C levels. **e, f** The mRNA levels of KIM-1 and NGAL. **g** Representative HE images of kidney. Arrows indicate injured tubules. **h** Analysis of tubular injury score in mice. **i** Representative western blots and quantitative analysis of NGAL and KIM-1. **j** The mRNA levels of IL-1β, IL-6, MCP-1, and COX-2. **k** The levels of serum TNF-α and IL-6. **l** Representative images and quantitative analysis of TUNEL staining (Scale bar = 100 μm). **m** Representative images and quantitative analysis of DHE staining (Scale bar = 100 μm). ∗P < 0.05, ∗∗P < 0.01, ∗∗∗P < 0.001 versus the indicated group.

As stated above, *P. goldsteinii* holds promise as a potential probiotic for renal IRI therapy. Within a small molecule pool comprising 79 compounds, we screened for candidates that could promote the growth of *P. goldsteinii* at various time points (Fig. S11a). Our results revealed that Hamaudol exhibited the most potent ability to enhance the growth of *P. goldsteinii* (Fig. S11b). Consequently, Hamaudol supplementation increased the abundance of *P. goldsteinii*, particularly under CR conditions (Fig. S11c). Subsequently, we assessed whether Hamaudol could confer protective effects against ischemic kidney injury (Fig. 8a). Hamaudol was unable to prevent CR-induced body weight loss in mice (Table S10). In spite of this, serum levels of BUN, Scr, and cystatin C were elevated in IRI-induced mice, whereas Hamaudol administration ameliorated these abnormalities (Fig. 8b–d). Histological analysis, along with tubular injury markers from HE staining, qPCR, and western blotting, demonstrated that Hamaudol mitigated histological lesions and renal tubule injury (Fig. 8e–i). Moreover, Hamaudol attenuated renal inflammation, apoptosis, and oxidative stress in renal IRI-induced mice (Fig. 8j–m). Similarly, we observed that renal IRI-induced intestinal barrier impairment, occludin and ZO-1 downregulations, as well as LPS elevations, were alleviated by Hamaudol treatment (Figs. S2a and S12). Notably, these beneficial effects of Hamaudol were further enhanced when combined with CR treatment (Fig. 8). Taken together, our findings suggest that Hamaudol can mitigate renal damage by promoting the growth of *P. goldsteinii*. Eventually, we found that CR, LPG, dodecafluorpentan, and Hamaudol exhibited similar reno-protective effects by improving renal function, mitigating post-ischemic renal pathological changes, and inhibiting the release of IL-6 at 7 days post-injury (Fig. S13).

## 4 Discussion

**Fig. 9:**
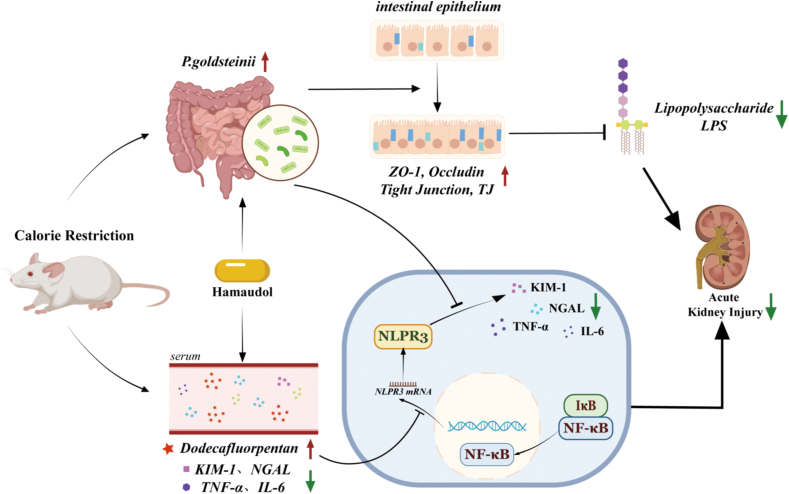
A sketch diagram showing the benefits and mechanisms of CR in preventing and treating AKI.

A growing number of studies have recognized the contribution of gut microbiota and metabolites to human health and diseases. Preconditioning with CR is highly protective against renal insult in AKI rodents [14,15,38,39]. It remains to be answered whether CR confers a protection against AKI through mechanisms dependent on the gut microbiota. In this study, we observed significantly reduced renal damage after CR treatment, and gut microbiome depletion abolished the actions of CR on the kidneys. In keeping with this, mice receiving microbiota transplants from CR mice showed resistance to renal injury induced by AKI. 16S rRNA gene sequencing showed that CR enriched the abundance of *P. goldsteinii* in the gut, and oral administration of *P. goldsteinii* improved renal dysfunction and renal tubular damage in mice with renal IRI, akin to CR. CR upregulated the contents of a metabolite dodecafluorpentan to induce a protection against AKI since application of dodecafluorpentan significantly reduced the pathological changes of kidneys and ameliorated the intestinal barrier disorder in AKI mice. High throughput transcriptomics suggested that dodecafluorpentan protected against AKI by suppressing the nuclear translocation of NFκB and inhibiting the subsequent activation of NLRP3 inflammasome in renal tubular epithelial cells. Eventually, we identified that Hamaudol had obvious protective effects on AKI by potentiating the growth of *P. goldsteinii*, thus Hamaudol may have the potential to be a candidate drug for the treatment of AKI by regulating gut bacterial flora and repairing the intestinal barrier (Fig. 9).

Microbiota-modifying therapies might lead to novel approaches for the prevention and treatment of AKI. The loss of gut flora richness and biodiversity is intimately linked to various disorders, including AKI. Targeting gut microbiota presents a potential avenue for treating or managing experimental AKI. Treating ischemic AKI mice with amoxicillin significantly restored renal function recovery and prevented the progression of AKI to CKD by reshaping the gut bacteria, such as increased *Alistipes*, *Odoribacter*, and *Stomatobaculum* species, and depleted *Holdemanella* and *Anaeroplasma* [40]. Increased *Enterobacteriacea* and decreased *Lactobacilli* and *Ruminococacceae* are the hallmarks of renal IRI, and colonizing germ-free mice with post-AKI microbiota aggravated renal IRI in recipient mice [1]. These findings collectively underscore the potential therapeutic strategies for AKI by targeting the intestinal microbiota. Accumulative evidence supports CR as a promising therapeutic approach for AKI. Given its potential as an AKI therapy, the usage of CR warrants special attention [41]. However, whether the renal benefits of CR in AKI are contingent upon gut microbiota regulation remains largely unknown. In this study, we found that gut microbiota was responsible for the protective effects of CR in renal dysfunction post-IRI, as oral antibiotics markedly nullified the effects of CR on renal damage induced by IRI. Colonization of germ-free mice with CR-derived microbiota ameliorated renal injury in response to AKI, suggesing that CR-induced renal benefits were largely dependent on gut microbiota. Further studies showed that CR enhanced the abundance of *P. goldsteinii* in renal IRI mice. Importantly, the abundance of fecal *P. goldsteinii* was lower in AKI patients, and this decrease was conversely associated with Scr levels, indicating that *P. goldsteinii* may serve as a potential probiotic in AKI. Actually, administration of *P. goldsteinii* showed protective effects against AKI by ameliorating perturbations in BUN, Scr and cystatin C levels, as well as attenuating the pathological changes in kidneys.

Previously, it was reported that treating mice fed a high-fat diet with LPG reduced obesity and increased adipose tissue thermogenesis, improved intestinal integrity, and decreased inflammation and insulin resistance [17]. Gavage with *P. goldsteinii* improved surgical colonic healing by exerting an anti-inflammatory effect in patients with colorectal cancer [42]. Treatment with *P. goldsteinii* significantly decreased both intestinal and systemic inflammation and improved autism relevant behaviors induced by maternal immune activation [43]. The commensal bacterium *P. goldsteinii* was found to improve chronic obstructive pulmonary disease (COPD) in mice by yielding the anti-inflammatory LPS [18]. Mice supplemented with *P. goldsteinii* are insensitive to aspirin-mediated damage of the intestinal niche [35]. Increased *P. goldsteinii* level plays a beneficial role in metabolic disorders [44]. Depleted probiotic *P. goldsteinii* contributes to tumorigenesis of male colorectal cancer mice [45]. It is likely that *P. goldsteinii* may be developed as promising probiotic for various human diseases. In the present study, administration of *P. goldsteinii* exerted renal benefits in AKI mice, akin to CR. The gut microbiome functions as an endocrine organ by producing bioactive metabolites such as SCFAs and bile acids that can influence host physiology. However, it remains to be answered how *P. goldsteinii* protected against AKI. The key metabolites produced by *P. goldsteinii* and their metabolic enzymes require future identification, thus enhancing our understanding of therapeutic potential and mechanisms of *P. goldsteinii* in AKI.

Through untargeted and targeted metabolomics, we found that serum levels of dodecafluorpentan were significantly diminished in AKI mice, which was lifted by CR. However, the specific role of this metabolite in kidney protection during AKI remained unclear, as did its pharmacological effects on different systems. To address this, we examined the potential role of dodecafluorpentan in AKI pathogenesis, and found that dodecafluorpentan protected against renal tubular epithelial cell injury *in vitro* and *in vivo*. The NLRP3 inflammasome is made up of the NLRP3 sensor, ASC adaptor, and caspase-1 protease. Activation of the NLRP3 inflammasome leads to the maturation and secretion of IL-1β through the activation of caspase-1. Additionally, activation of the NLPR-3/caspase-1/IL-1β inflammasome pathway is a core event contributing to AKI development and progression [46]. Toll-like receptor 4 (TLR4) has been recognized as a crucial regulator of inflammatory pathways, playing a significant role in the development of AKI. The interaction between LPS and TLR4 triggers the activation of the transcription factor NF-κB. Upon activation, NF-κB translocates to the nucleus, regulating immune and inflammatory responses by releasing inflammatory mediators like IL-1β and IL-18 via transcriptionally activating NLRP3 inflammasome. Our RNA sequencing and experimental data revealed that dodecafluorpentan protected against AKI by antagonizing NFκB-induced NLRP3 inflammasome activation. These results indicated that dodecafluorpentan could confer benefit for treating AKI. While promising, it remains unclear if dodecafluorpentan primarily originates from *P. goldsteinii*, necessitating further evidence. It is essential to recognize that serum metabolites may play critical roles in individual physiological stress, disease progression, and drug development, making their application in AKI a burgeoning field. Extensive studies are required to fully explore the potential of dodecafluorpentan as a candidate drug for AKI treatment.

In light of the interest of *P. goldsteinii* in AKI therapy, our drug screening experiments showed that Hamaudol ameliorated renal insufficiency in renal IRI mice by promoting the growth of *P. goldsteinii*. It is likely that Hamaudol may be an attractive prebiotic with protective effects against AKI, this deserved more studies. Overall, our study unveiled the role of gut microbiota and metabolites in mediating the protective effects of CR following AKI, as indicated by 16S rRNA sequencing analysis, antibiotics administration, FMT, metabolomics, and transcriptomics. We also identified that dodecafluorpentan protected against renal dysfunction post-IRI through inhibiting NFκB and NLRP3 inflammasome pathways. It is interesting to investigate the breadth of AKI which is known to have an injury peak around 7 days. Thereafter, we examined the actions of CR, LPG, dodecafluorpentan, and Hamaudol on renal function after 7 days of renal IRI. Results showed that CR, LPG, dodecafluorpentan, and Hamaudol led to a significant alleviation of renal dysfunction at 7 days post-injury. However, more experiments are recommended to investigate the protective effects and mechanisms of CR, LPG, dodecafluorpentan, and Hamaudol in improving renal injury peak in AKI mice. Our results provided insights into the compositions of the gut microbiota in CR mice and enhanced our understanding of the effect of dodecafluorpentan on renal IRI, thus linking CR, microbiome, and metabolites with AKI.

## Limitations

Our present study had several limitations that warrant consideration. Firstly, we did not assess whether long-term CR improved AKI by modulating gut microbiota. Secondly, the impact of short-term CR on the progression of AKI to CKD via gut microbiota modulation remains unexplored. Thirdly, the precise mechanism underlying the renal protective effects of *P. goldsteinii* remains unclear due to a lack of identification of the exact metabolites produced by *P. goldsteinii*. Fourthly, only male mice were used in this study, future studies are warranted to include both genders to comprehensively understand the gender-specific effects of gut microbiome and caloric restriction on AKI. Fifthly, it is important to distinguish the effects of pre-treatment from those of interventions administered at the time of injury. Future studies are highly required to conduct additional experiments where the dietary changes and therapeutics are administered at the time of injury rather than as pre-treatment. This new experimental approach aims to assess the efficacy of these interventions in mitigating AKI when initiated post-injury. The comparisons between pre-treatment and post-injury intervention groups will help to clarify the contributions of weight loss and other changes associated with the interventions. Most importantly, a tweak in the model where injury is induced and dietary changes or therapeutics are given at the time of injury would be very informative and lead to broader appeal and utilization of our current findings. Sixthly, we only found diminished levels of *P. goldsteinii* and dodecafluorpentan in AKI patients, whether they play a preventive and therapeutic role in AKI patients still needs to be studied. Additionally, the origin of serum metabolite dodecafluorpentan is ambiguous, and whether *P. goldsteinii* produced dodecafluorpentan is yet to be determined. Lastly, it is deserved to investigate whether Hamaudol exerts renal protective effects through modulation of other gut microbiota, not only *P. goldsteinii*.

## Funding

This work was supported by the 10.13039/501100001809National Natural Science Foundation of China (82300414, 823700364, 8217021262 and 81700364), Jiangsu Province Excellent Youth Fund (BK20240204), 10.13039/501100004608Jiangsu Natural Science Foundation (BK20231049, BK20170179, BE2020634 and BK20191138), high-level introduction of talents and scientific research start-up funds of 10.13039/501100004024JNU (1286010241230530), the 10.13039/501100012226Fundamental Research Funds for the Central Universities (JUSRP124036), 10.13039/501100004608Jiangsu Province department of science and technology (BE2020634, BK20191138), Top Talent Support Program for young and middle-aged people of Wuxi Health Committee (BJ2020049) and Clinical Research and Translational Medicine Research Program of Affiliated Hospital of Jiangnan University (LCYJ202306, LCYJ202226), Wuxi Science and Technology Development Fund Project "Light of the Taihu Lake"(K20221028), Wuxi Municipal Health Commission Youth Project (Q202226), Wuxi City "Double Hundred" Young and Middle aged Medical and Health Top Talents Training Program Project (HB2023045), the Translational Medicine Project of the Wuxi Municipal Health Commission [ZH202107], Medical Discipline Program of Wuxi Health Commission, the Science and Technology Projects of Wuxi City (M202207, BJ2023029).

## Availability of data and materials

The 16S rRNA gene sequencing data have been deposited in the NCBI Sequence Read Archive (SRA) database under accession code PRJNA1090669.

Raw RNA sequencing data have been deposited in the Sequencing Read Archive (SRA) under accession number PRJNA1091143.

## Ethics approval and consent to participate

All experimental procedures were conducted with approval from the Jiangnan University Institutional Animal Care and Use Committee (JN.No20231007m0960430[473]) and the criteria in the Guide for the Care and Use of Laboratory Animals published by the US National Institutes of Health.

This study was adhered to the ethical principles outlined in the 1975 Declaration of Helsinki and received prior approval from the Ethics Committee of Nanjing Medical University (20180986-K041).

## Consent for publication

Not applicable.

## CRediT authorship contribution statement

**Xue-Xue Zhu:** Conceptualization, Funding acquisition, Writing – original draft, Writing – review & editing. **Xiao Fu:** Formal analysis, Methodology, Project administration. **Xin-Yu Meng:** Formal analysis, Methodology, Project administration. **Jia-Bao Su:** Formal analysis, Methodology, Project administration. **Guan-Li Zheng:** Investigation, Software, Visualization. **An-Jing Xu:** Investigation, Software, Visualization. **Guo Chen:** Investigation, Software, Visualization. **Yuan Zhang:** Investigation, Software, Visualization. **Yao Liu:** Investigation, Software, Visualization. **Xiao-Hui Hou:** Investigation, Software, Visualization. **Hong-Bo Qiu:** Investigation, Software, Visualization. **Qing-Yi Sun:** Investigation, Software, Visualization. **Jin-Yi Hu:** Investigation, Software, Visualization. **Zhuo-Lin Lv:** Investigation, Software, Visualization. **Yao Wang:** Investigation, Software, Visualization. **Hai-Bin Jiang:** Investigation, Software, Visualization. **Neng Bao:** Conceptualization, Funding acquisition, Writing – original draft, Writing – review & editing. **Zhi-Jun Han:** Conceptualization, Funding acquisition, Writing – original draft, Writing – review & editing. **Qing-Bo Lu:** Conceptualization, Funding acquisition, Writing – original draft, Writing – review & editing. **Hai-Jian Sun:** Conceptualization, Funding acquisition, Writing – original draft, Writing – review & editing.

## Declaration of competing interest

None.

## References

[1] Yang J., Kim C.J., Go Y.S., Lee H.Y., Kim M.G., Oh S.W. (2020). Intestinal microbiota control acute kidney injury severity by immune modulation. Kidney Int..

[2] Yang L., Xing G., Wang L., Wu Y., Li S., Xu G. (2015). Acute kidney injury in China: a cross-sectional survey. Lancet (London, England).

[3] Koyner J.L. (2021). Sepsis and kidney injury. Contrib. Nephrol..

[4] Poston J.T., Koyner J.L. (2019). Sepsis associated acute kidney injury. BMJ.

[5] Kalim S., Rhee E.P. (2017). An overview of renal metabolomics. Kidney Int..

[6] Wei Q., Xiao X., Fogle P., Dong Z. (2014). Changes in metabolic profiles during acute kidney injury and recovery following ischemia/reperfusion. PLoS One.

[7] Gong J., Noel S., Pluznick J.L., Hamad A.R.A., Rabb H. (2019). Gut microbiota-kidney cross-talk in acute kidney injury. Semin. Nephrol..

[8] Chou Y.T., Kan W.C., Shiao C.C. (2022).

[9] Haro C., Mónaco M.E., Medina M. (2018). Lactobacillus casei beneficially modulates immuno-coagulative response in an endotoxemia model. Blood Coagul. Fibrinolysis : an international journal in haemostasis and thrombosis.

[10] Iwata Y., Nakade Y., Kitajima S., Yoneda-Nakagawa S., Oshima M., Sakai N. (2022).

[11] Nakade Y., Iwata Y., Furuichi K., Mita M., Hamase K., Konno R. (2018). Gut microbiota-derived D-serine protects against acute kidney injury. JCI insight.

[12] Mattison J.A., Roth G.S., Beasley T.M., Tilmont E.M., Handy A.M., Herbert R.L. (2012). Impact of caloric restriction on health and survival in rhesus monkeys from the NIA study. Nature.

[13] Mitchell J.R., Verweij M., Brand K., van de Ven M., Goemaere N., van den Engel S. (2010). Short-term dietary restriction and fasting precondition against ischemia reperfusion injury in mice. Aging Cell.

[14] Johnsen M., Kubacki T., Yeroslaviz A. (2020). The integrated RNA landscape of renal preconditioning against ischemia-reperfusion. Injury.

[15] Späth M.R., Bartram M.P., Palacio-Escat N., Hoyer K.J.R., Debes C., Demir F. (2019). The proteome microenvironment determines the protective effect of preconditioning in cisplatin-induced acute kidney injury. Kidney Int..

[16] Koehler F.C., Späth M.R., Hoyer-Allo K.J.R., Müller R.U. (2022). Mechanisms of caloric restriction-mediated stress-resistance in acute kidney injury. Nephron.

[17] Wu T.R., Lin C.S., Chang C.J., Lin T.L., Martel J., Ko Y.F. (2019). Gut commensal Parabacteroides goldsteinii plays a predominant role in the anti-obesity effects of polysaccharides isolated from Hirsutella sinensis. Gut.

[18] Lai H.C., Lin T.L., Chen T.W., Kuo Y.L., Chang C.J., Wu T.R. (2022).

[19] Okuyama E., Hasegawa T., Matsushita T., Fujimoto H., Ishibashi M., Yamazaki M. (2001). Analgesic components of saposhnikovia root (Saposhnikovia divaricata). Chem. Pharmaceut. Bull..

[20] Tang D., Wang C., Gu Z., Li J., Jin L., Li J. (2023). Discovery of anti-allergic components in Guomingkang Formula using sensitive HEMT biochips coupled with in vitro and in vivo validation. Phytomedicine : international journal of phytotherapy and phytopharmacology.

[21] Xu X., Yan S., Zhang Y., Cao L., Chen T., Yang X. (2024). Comparison of the chemical constituents of Saposhnikoviae Radix associated with three different growth patterns and its therapeutic effect against atopic dermatitis. J. Ethnopharmacol..

[22] Zhang N., Li Z., Mu W., Li L., Liang Y., Lu M. (2016). Calorie restriction-induced SIRT6 activation delays aging by suppressing NF-κB signaling. Cell Cycle.

[23] Li X., Yuan F., Xiong Y., Tang Y., Li Z., Ai J. (2024). FAM3A plays a key role in protecting against tubular cell pyroptosis and acute kidney injury. Redox Biol..

[24] Li Y., Dong B., Wang Y., Bi H., Zhang J., Ding C. (2024). Inhibition of Usp14 ameliorates renal ischemia-reperfusion injury by reducing Tfap2a stabilization and facilitating mitophagy. Transl. Res. : J. Lab. Clin. Med..

[25] Ma S., Wang D.H. (2021). Knockout of Trpa1 exacerbates renal ischemia-reperfusion injury with classical activation of macrophages. Am. J. Hypertens..

[26] Zhou Y., Wang D., Li H., Pan Y., Xiang X., Wu Y. (2022). Association of acute kidney disease with the prognosis of ischemic stroke in the Third China National Stroke Registry. BMC Nephrol..

[27] Sun H., Peng J., Cai S., Nie Q., Li T., Kellum J.A. (2021). A translational study of Galectin-3 as an early biomarker and potential therapeutic target for ischemic-reperfusion induced acute kidney injury. J. Crit. Care.

[28] Lu Q.B., Du Q., Wang H.P., Tang Z.H., Wang Y.B., Sun H.J. (2020). Salusin-β mediates tubular cell apoptosis in acute kidney injury: involvement of the PKC/ROS signaling pathway. Redox Biol..

[29] Yu X., Meng X., Xu M., Zhang X., Zhang Y., Ding G. (2018). Celastrol ameliorates cisplatin nephrotoxicity by inhibiting NF-κB and improving mitochondrial function. EBioMedicine.

[30] Weidemann A., Bernhardt W.M., Klanke B., Daniel C., Buchholz B., Câmpean V. (2008). HIF activation protects from acute kidney injury. J. Am. Soc. Nephrol. : JASN (J. Am. Soc. Nephrol.).

[31] Sun H., Guo Y., Wang H., Yin A., Hu J., Yuan T. (2023).

[32] Xiao B., Xu Z., Viennois E., Zhang Y., Zhang Z., Zhang M. (2017). Orally targeted delivery of tripeptide KPV via hyaluronic acid-functionalized nanoparticles efficiently alleviates ulcerative colitis. Mol. Ther. : the journal of the American Society of Gene Therapy.

[33] Quan L.H., Zhang C., Dong M., Jiang J., Xu H., Yan C. (2020).

[34] Wang Q., Li W. (2024).

[35] Li T., Ding N., Guo H., Hua R., Lin Z., Tian H. (2024). A gut microbiota-bile acid axis promotes intestinal homeostasis upon aspirin-mediated damage. Cell Host Microbe.

[36] Lu Q.B., Fu X., Liu Y., Wang Z.C., Liu S.Y., Li Y.C. (2023).

[37] Sun H.J., Tan J.X., Shan X.D., Wang Z.C., Wu Z.Y., Bian J.S. (2023). DR region of NKAα1 is a target to ameliorate hepatic lipid metabolism disturbance in obese mice. Metab., Clin. Exp..

[38] Hoyer-Allo K.J.R., Späth M.R., Brodesser S., Zhu Y., Binz-Lotter J., Höhne M. (2022). Caloric restriction reduces the pro-inflammatory eicosanoid 20-hydroxyeicosatetraenoic acid to protect from acute kidney injury. Kidney Int..

[39] Beamish J.A., Telang A.C., McElliott M.C., Al-Suraimi A., Chowdhury M., Ference-Salo J.T. (2024). Pax protein depletion in proximal tubules triggers conserved mechanisms of resistance to acute ischemic kidney injury preventing transition to chronic kidney disease. Kidney Int..

[40] Gharaie S., Lee K., Newman-Rivera A.M., Xu J., Patel S.K., Gooya M. (2023). Microbiome modulation after severe acute kidney injury accelerates functional recovery and decreases kidney fibrosis. Kidney Int..

[41] Wang S.Y. (2018).

[42] Hajjar R., Gonzalez E., Fragoso G., Oliero M., Alaoui A.A., Calvé A. (2023).

[43] Lin T.L., Lu C.C., Chen T.W. (2022).

[44] Xu J., Tian H., Ji Y., Dong L., Liu Y., Wang Y. (2023). Urolithin C reveals anti-NAFLD potential via AMPK-ferroptosis axis and modulating gut microbiota. N. Schmied. Arch. Pharmacol..

[45] Wang L., Tu Y.X., Chen L., Zhang Y., Pan X.L., Yang S.Q. (2023). Male-biased gut microbiome and metabolites aggravate colorectal. Cancer Development.

[46] Saad H.M., Elekhnawy E., Shaldam M.A., Alqahtani M.J., Altwaijry N., Attallah N.G.M. (2024). Rosuvastatin and diosmetin inhibited the HSP70/TLR4/NF-κB p65/NLRP3 signaling pathways and switched macrophage to M2 phenotype in a rat model of acute kidney injury induced by cisplatin. Biomedicine & pharmacotherapy = Biomedecine & pharmacotherapie.

