# Supplementary material for: Gut microbiome and metabolites mediate the benefits of caloric restriction in mice after acute kidney injury
Source: Redox Biol. 2024 Sep 27;77:103373. doi: 10.1016/j.redox.2024.103373 (PMC11471245; doi:10.1016/j.redox.2024.103373)

Figure 1i

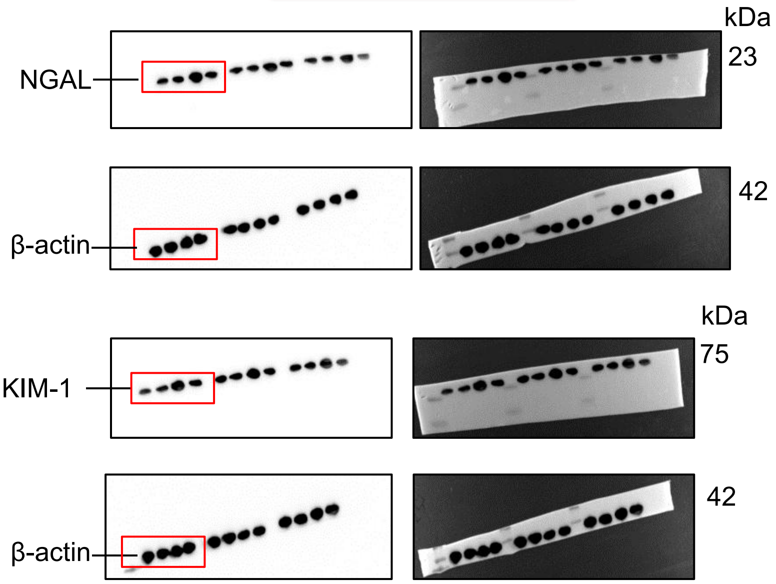

Figure 2i

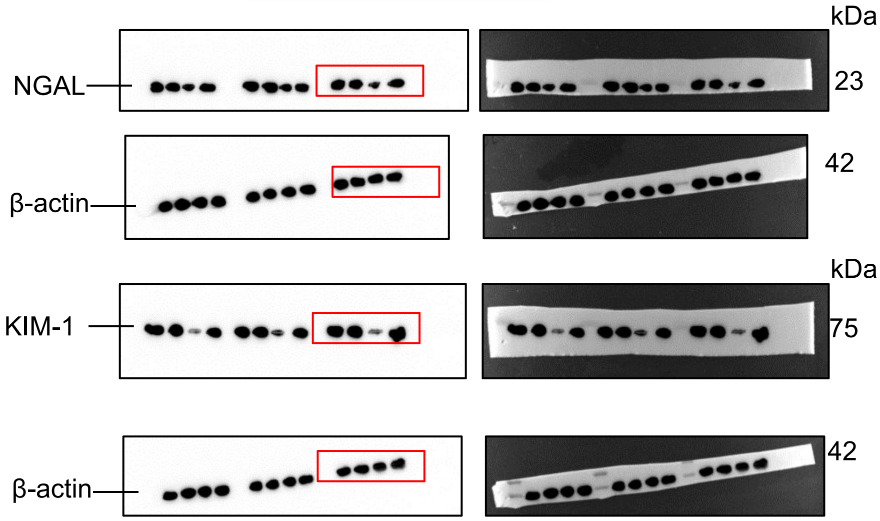

Figure 3i

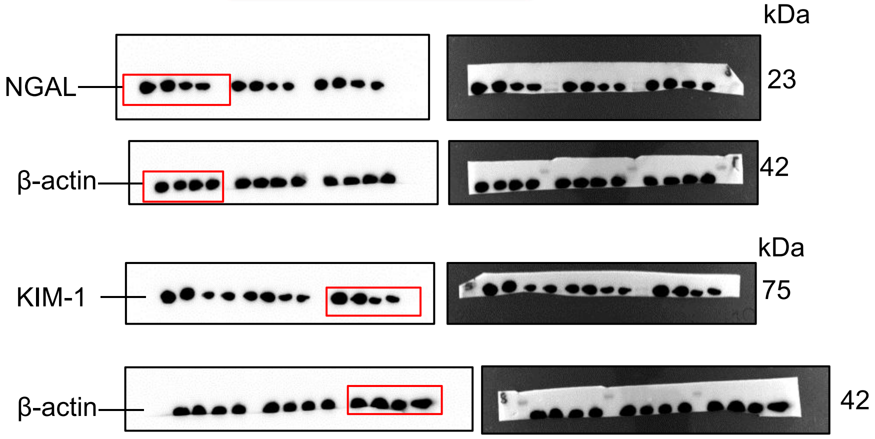

Figure 5h

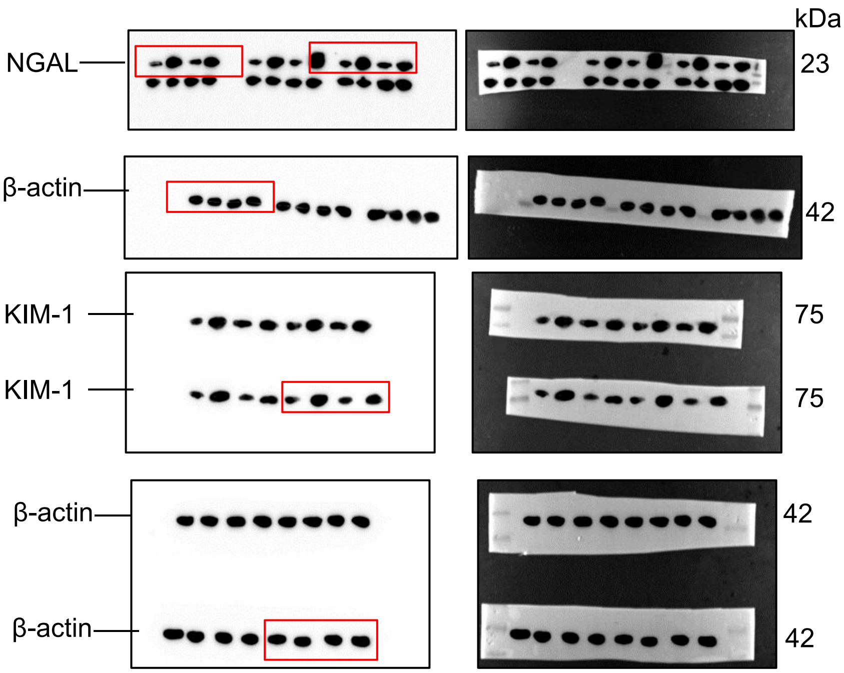

Figure 6m

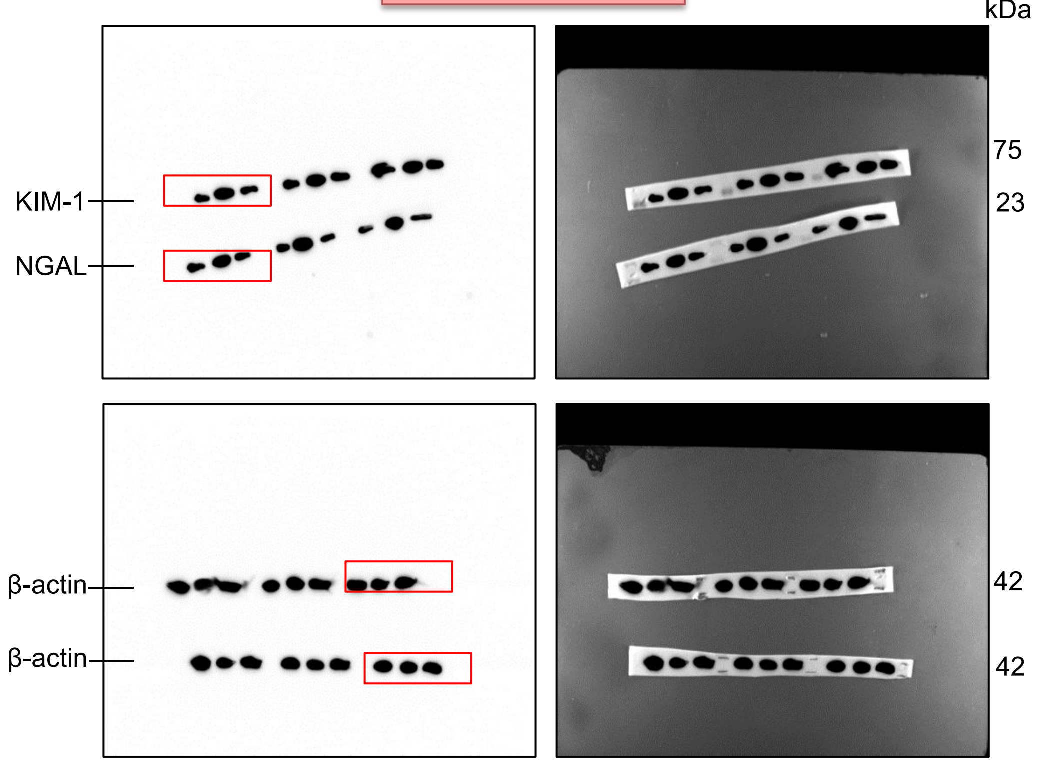

Figure 7a

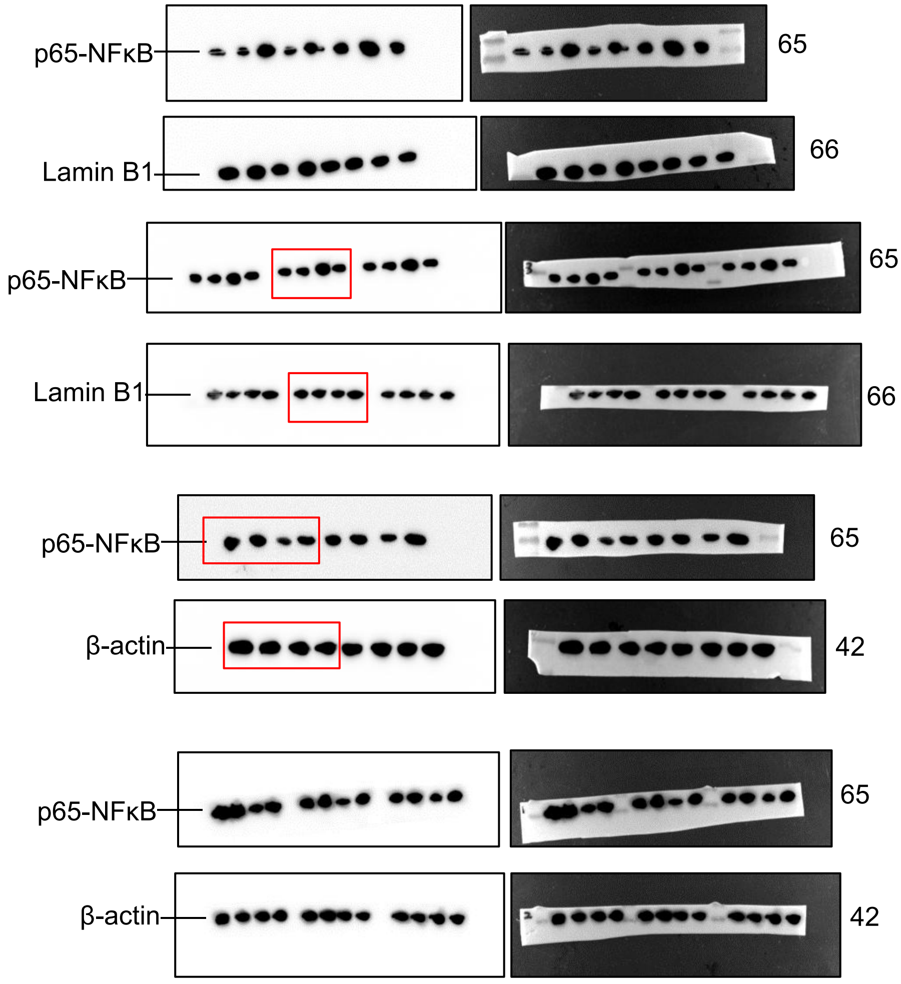

Figure 7c

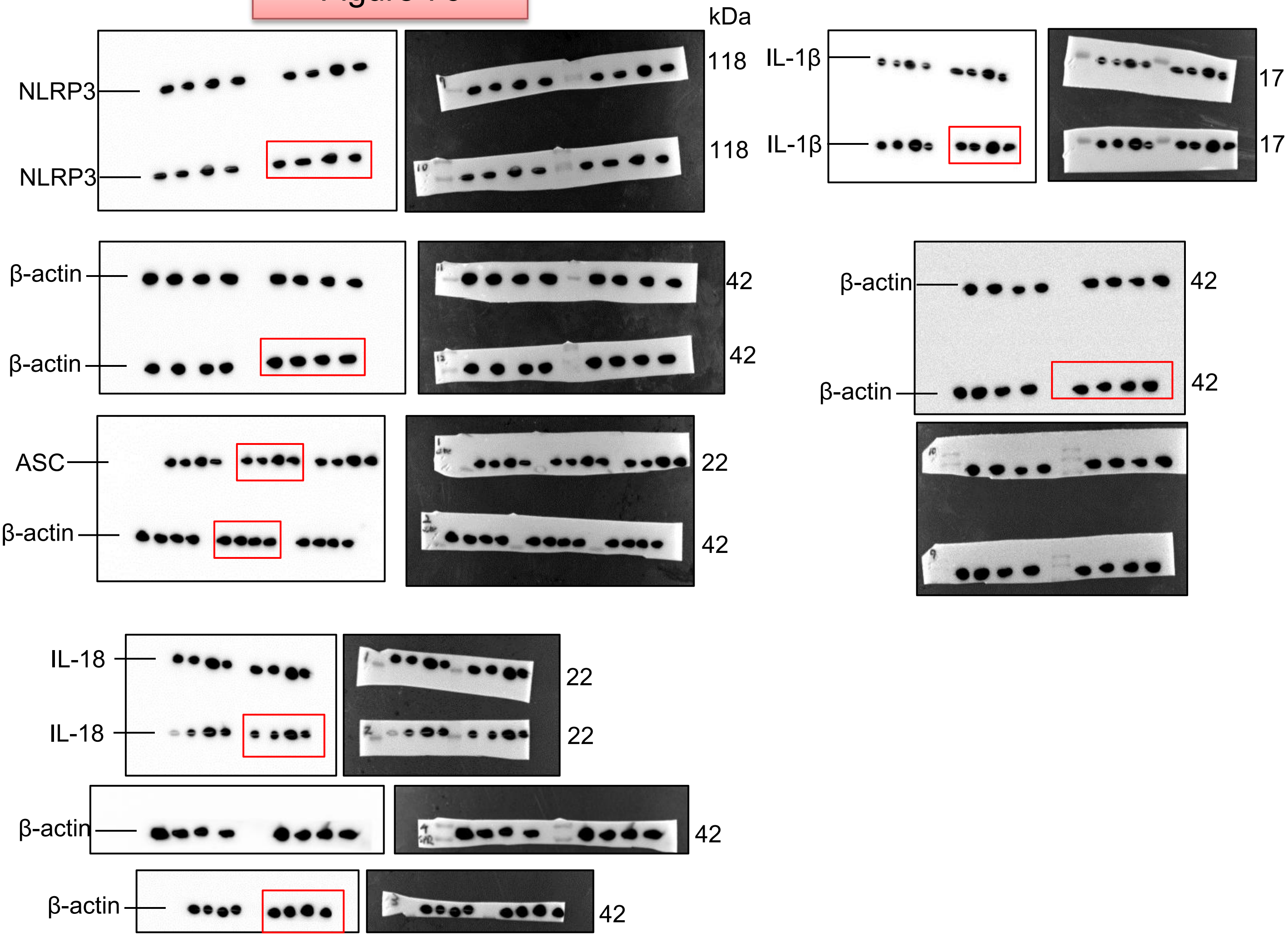

kDa
NLRP3
NLRP3
118
118
IL-1β
IL-1β
17
17
β-actin
β-actin
42
42
β-actin
β-actin
42
42
ASC
β-actin
22
42
IL-18
IL-18
22
22
β-actin
42
β-actin
42

Figure 7e

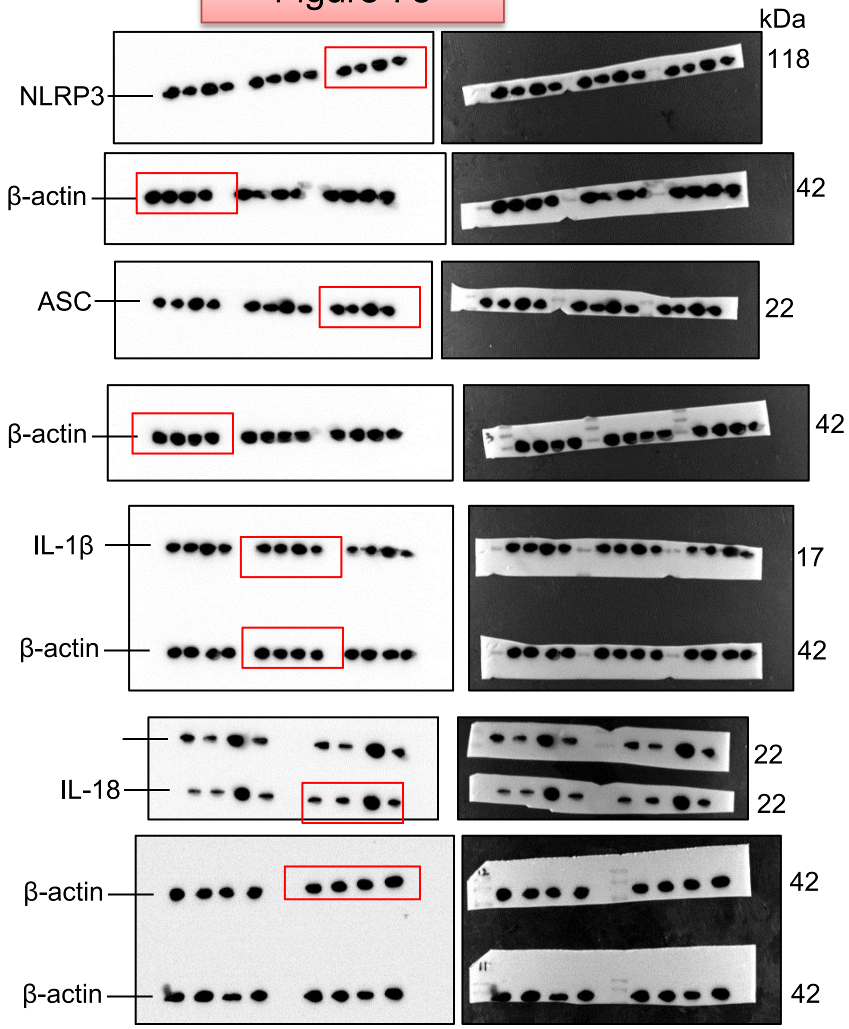
kDa
NLRP3
118
β-actin
42
ASC
22
β-actin
42
IL-1β
17
β-actin
42
22
IL-18
22
β-actin
42
β-actin
42

Figure 8i

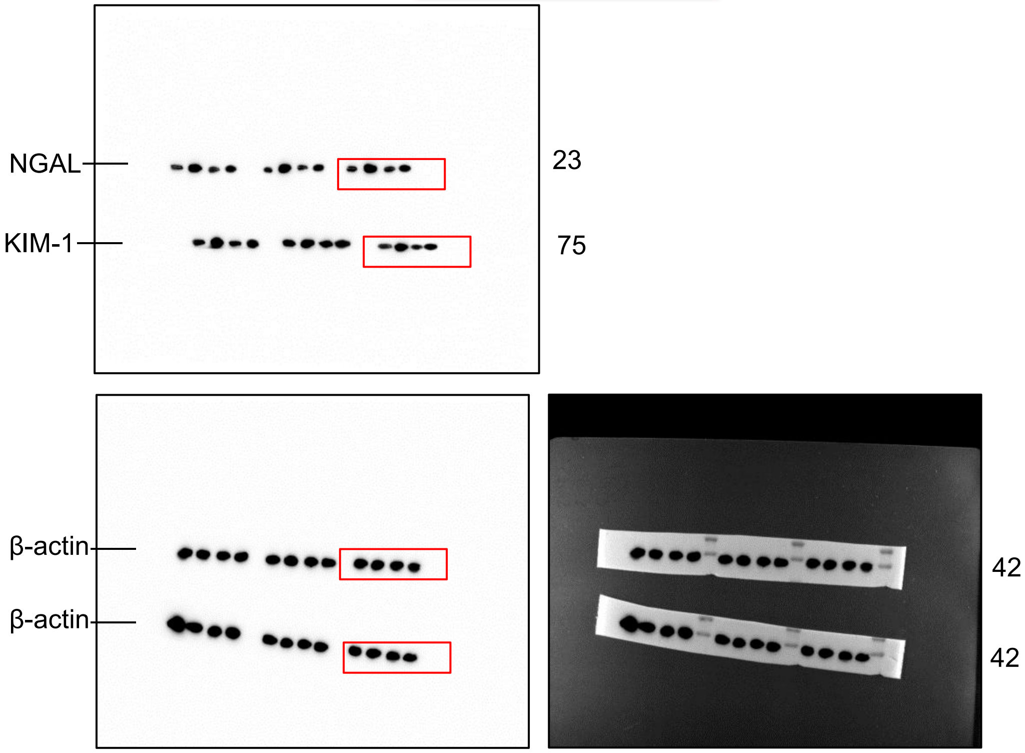

Figure S1c

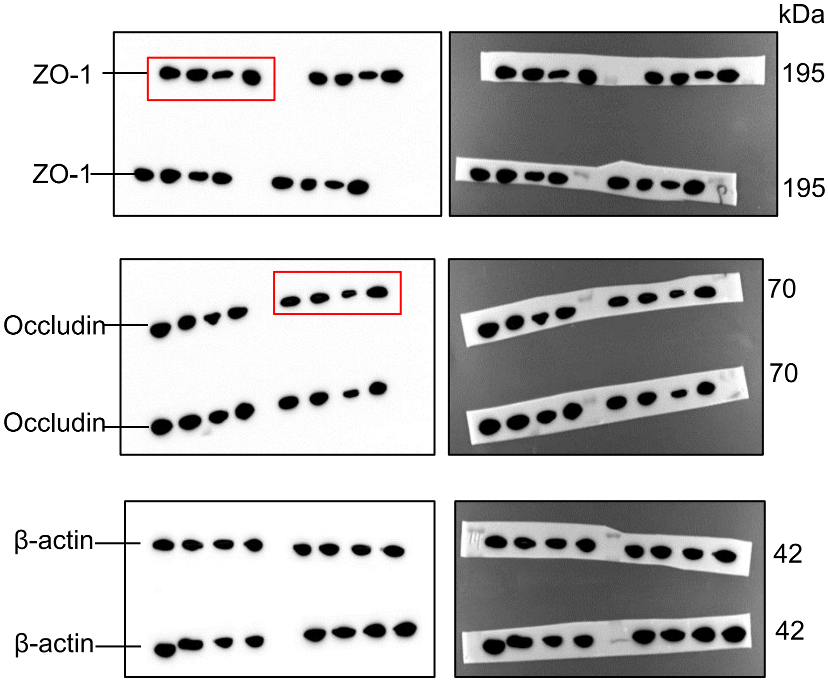

kDa
ZO-1
ZO-1
195
195
Occludin
Occludin
70
70
β-actin
β-actin
42
42

Figure S3h

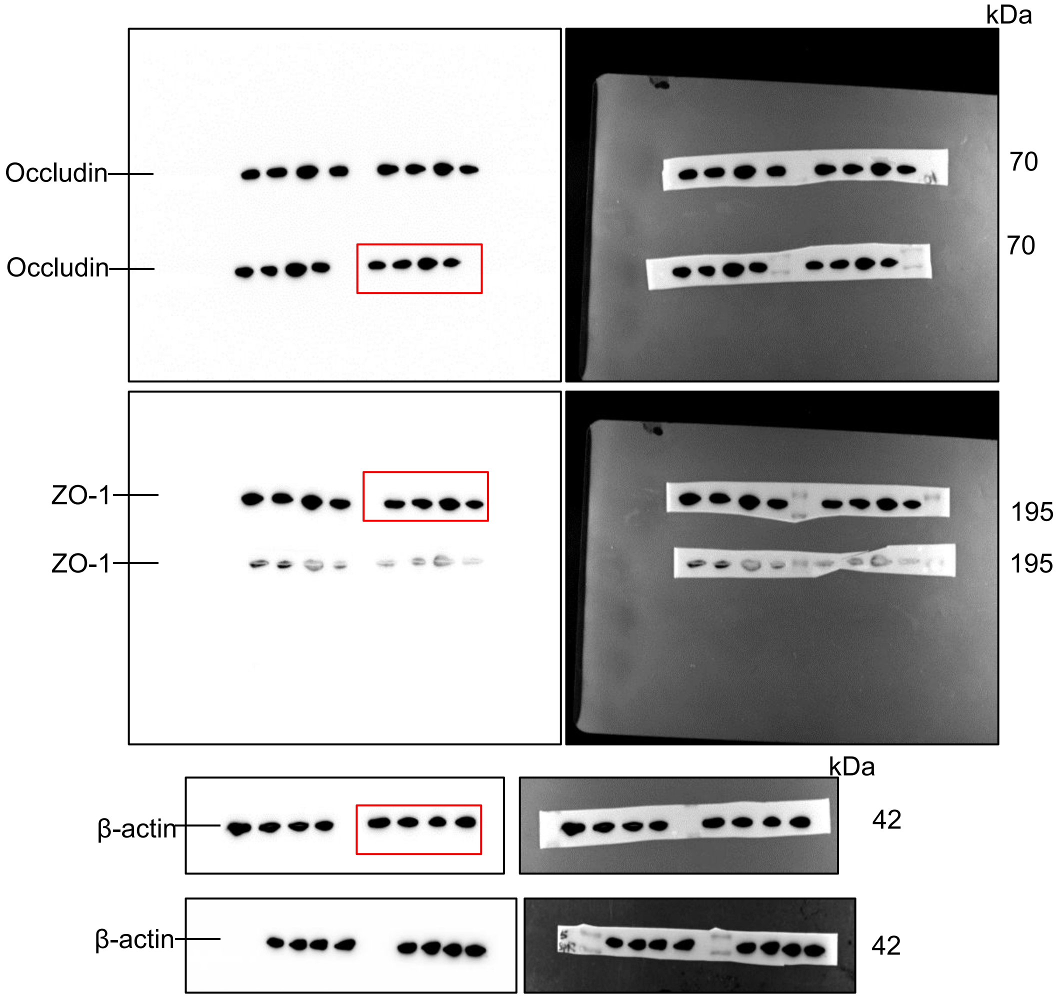

Figure S4g

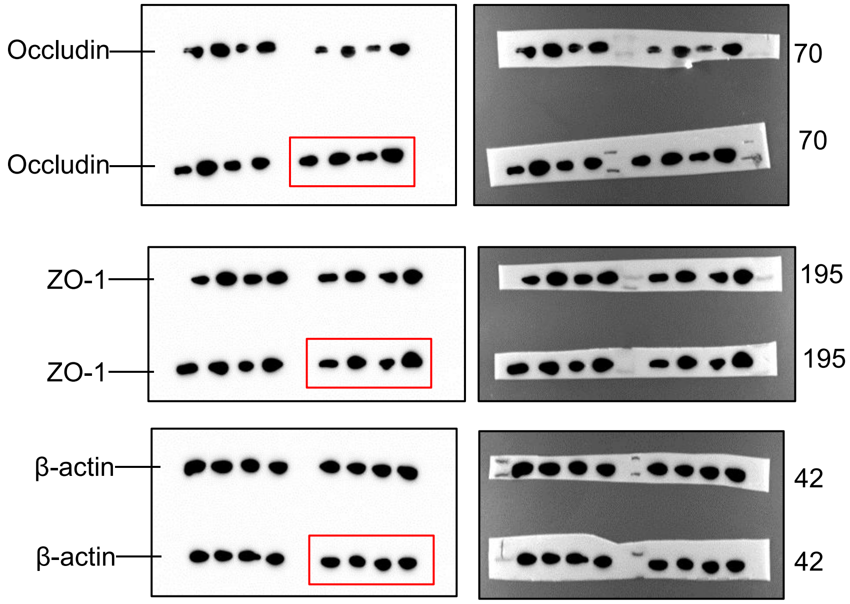

Figure S7g

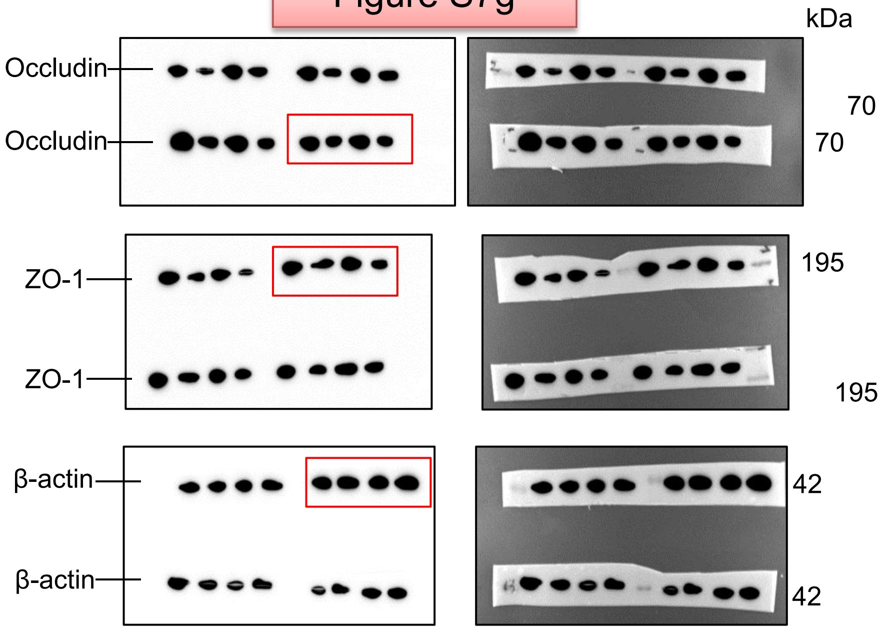

Figure S8g

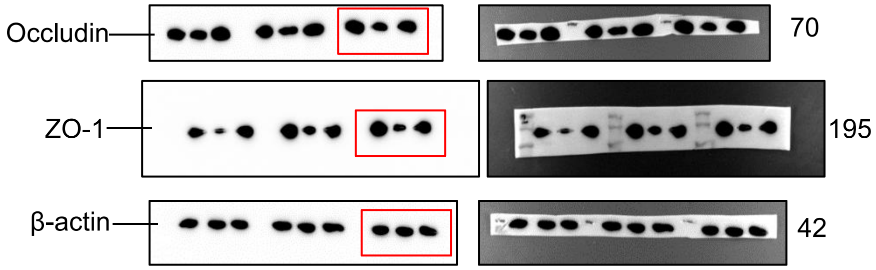

Figure S9j

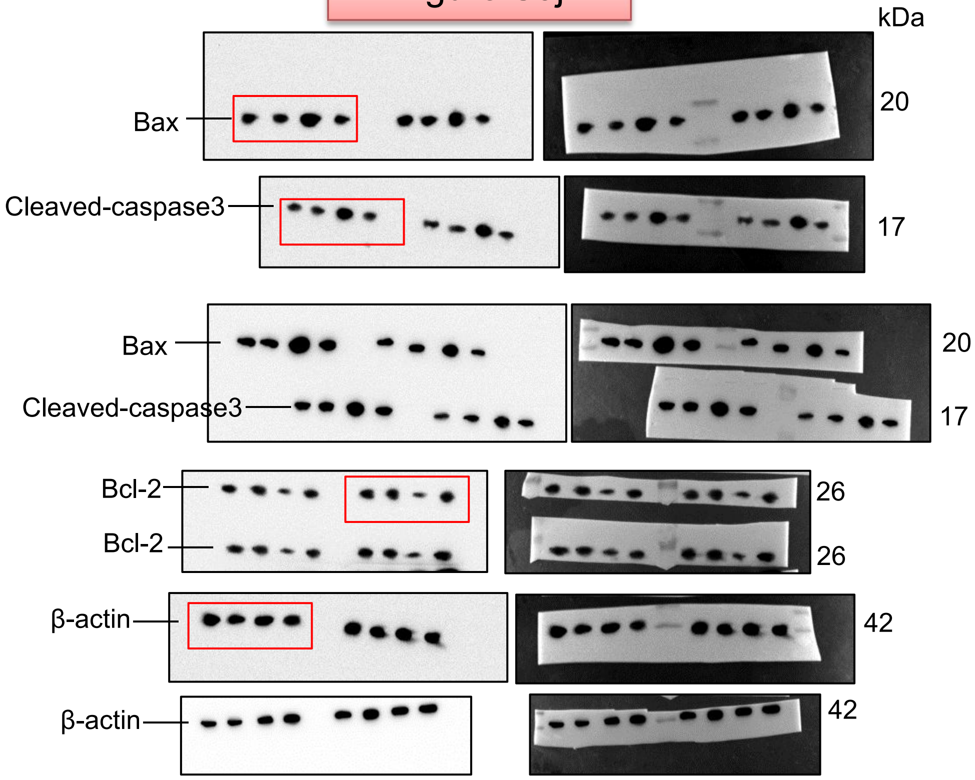

Figure S12g

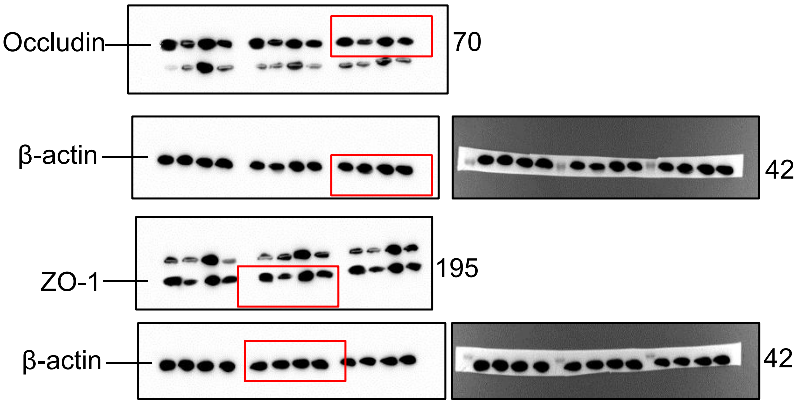

Supplement: Multimedia component 1 [file mmc1.pdf]
