# Supplementary material for: Gut microbiome and metabolites mediate the benefits of caloric restriction in mice after acute kidney injury
Source: Redox Biol. 2024 Sep 27;77:103373. doi: 10.1016/j.redox.2024.103373 (PMC11471245; doi:10.1016/j.redox.2024.103373)
Supplement: Multimedia component 2 [file mmc2.docx]

**Supplementary data**


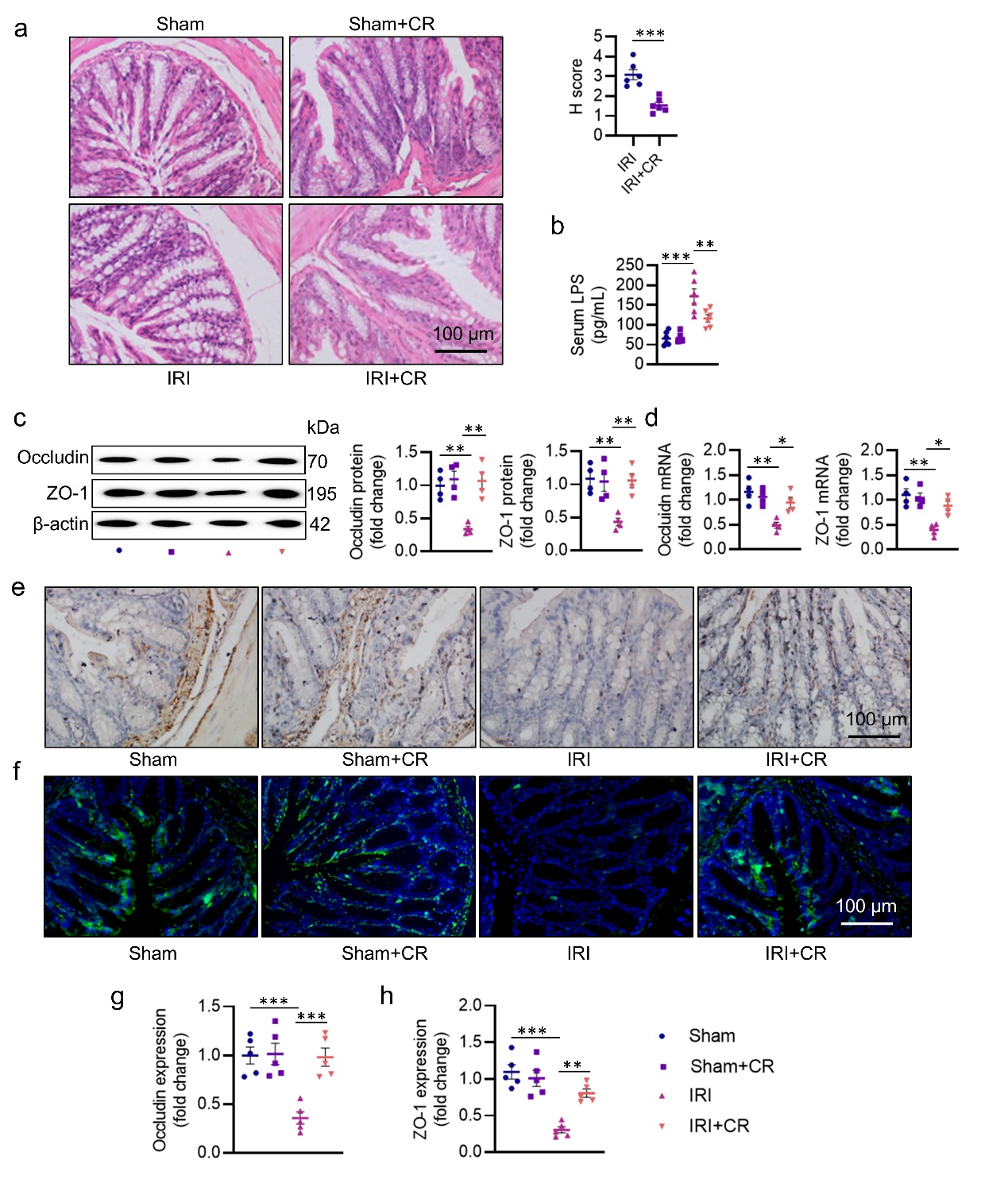


**Fig. S1. CR treatment attenuates IR-induced intestinal injury.** **a** Representative photographs and injury score of H&E staining in colon. **b** The release of serum LPS. **c** Representative western blots and quantitative analysis of Occludin and ZO-1. **d** The mRNA levels of Occludin and ZO-1. **e** Representative immunohistochemistry staining of Occludin in the colon. **f** Representative immunofluorescence staining of ZO-1 in the colon. **g** Relative analysis of Occludin. **h** Relative analysis of ZO-1. *P<0.05, **P<0.01, ***P<0.001 versus the indicated group.


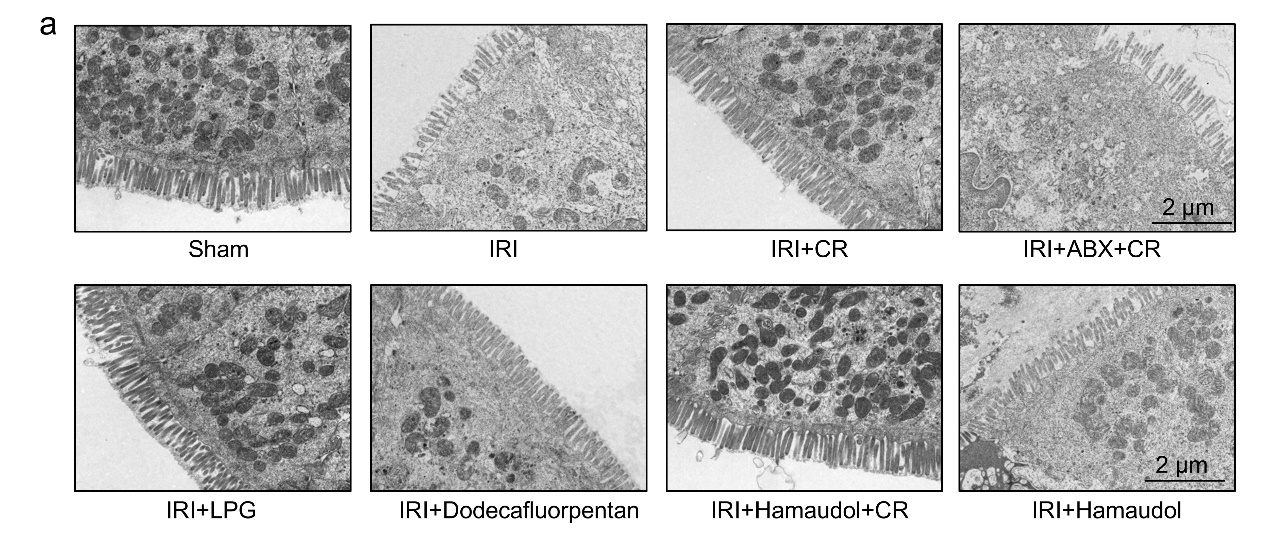


**Fig. S2.TEM images in the colon TJs and microvilli in different groups**.


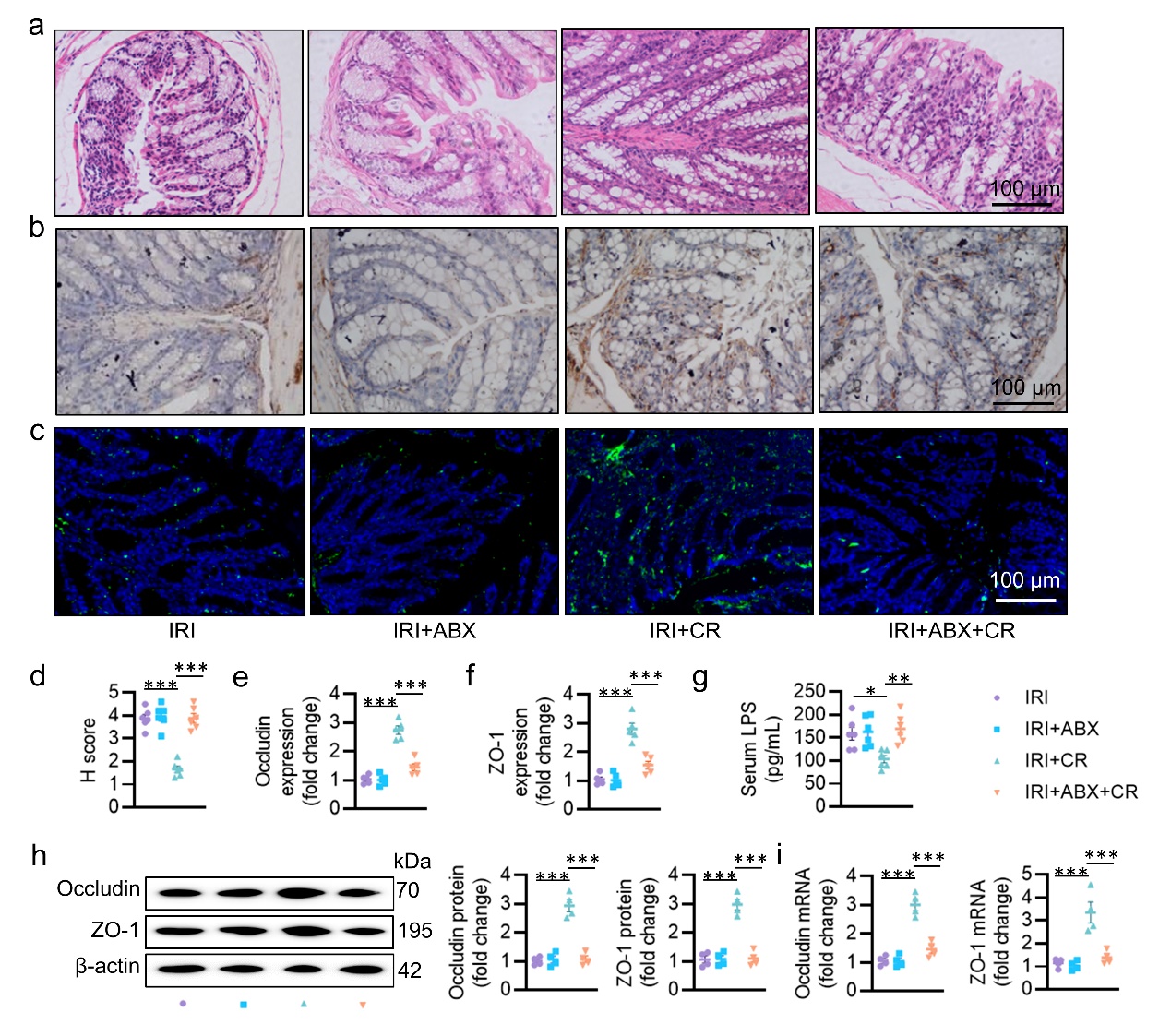


**Fig. S3. The effect of gut microbiota deletion on IR-induced intestinal injury. a** Representative H&E staining in colon. **b** Representative immunohistochemistry staining of Occludin in the colon. **c** Representative immunofluorescence staining of ZO-1 in the colon. **d** The injury score of colon. **e** Relative analysis of Occludin. **f** Relative analysis of ZO-1. **g** The release of serum LPS. **h** Representative western blots and quantitative analysis of Occludin and ZO-1. **i** The mRNA levels of Occludin and ZO-1. *P<0.05, **P<0.01, ***P<0.001 versus the indicated group.


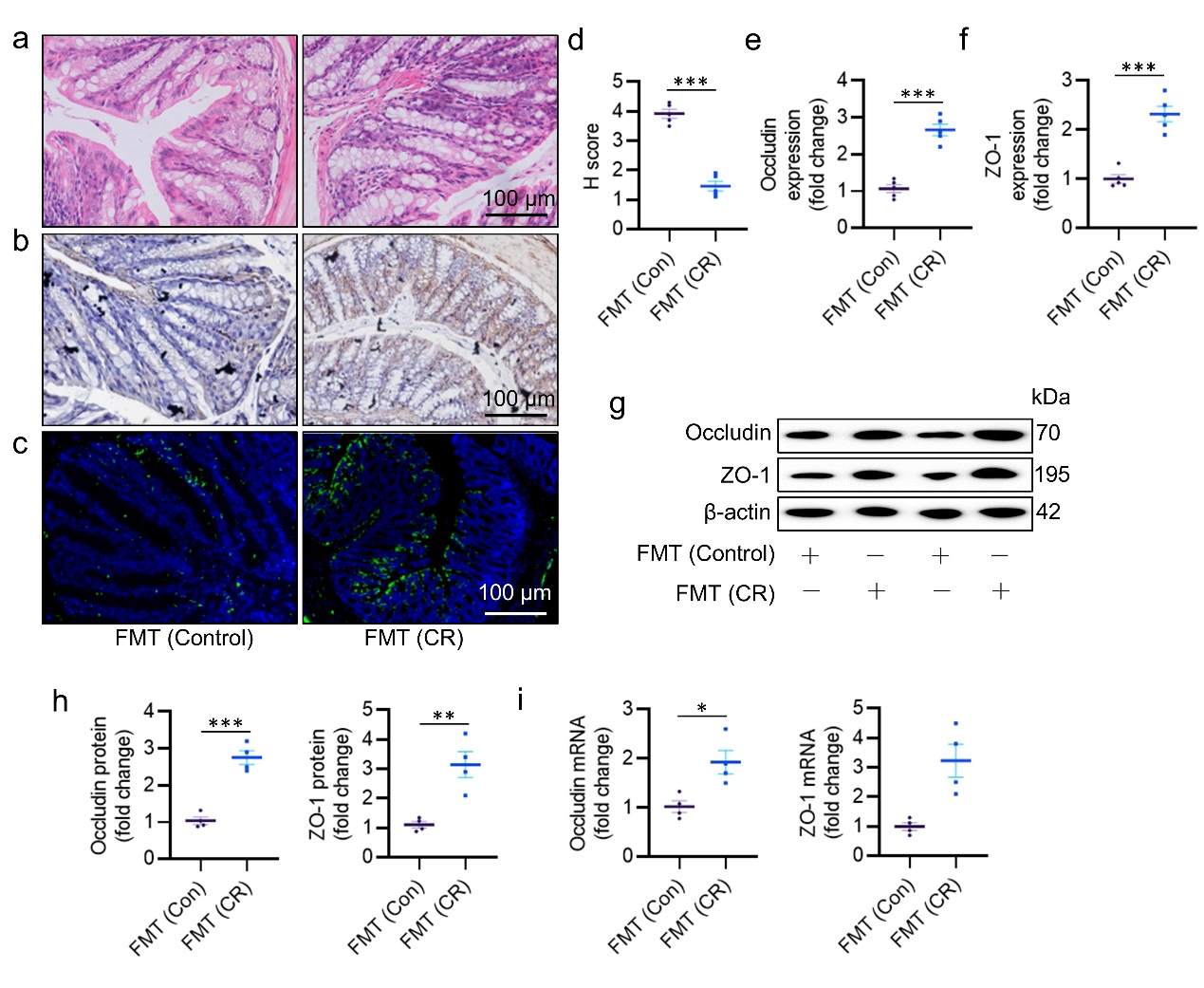


**Fig. S4. Effects of FMT on IR-induced intestinal damage. a** Representative photographs H&E staining in colon. **b** Representative immunohistochemistry staining of Occludin in the colon. **c** Representative immunofluorescence staining of ZO-1 in the colon. **d** The injury score of colon. **e** Relative analysis of Occludin. **f** Relative analysis of ZO-1. **g, h** Representative western blots and quantitative analysis of Occludin and ZO-1. **i** The mRNA levels of Occludin and ZO-1. *P<0.05, **P<0.01, ***P<0.001 versus the indicated group.


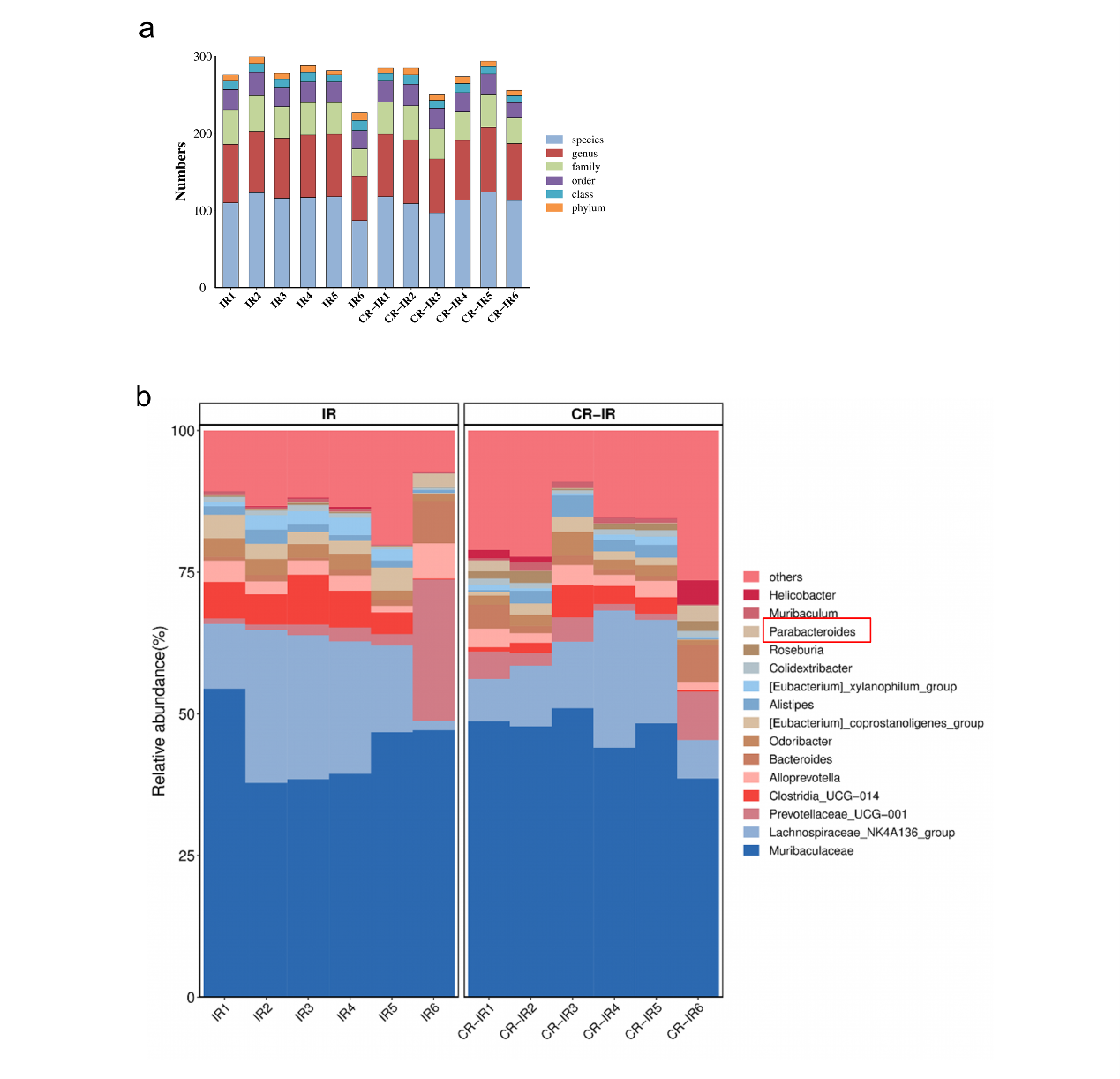


**Fig. S5. The effect of CR treatment on community diversity of gut microbiome in mice induced by IRI.** **a** The barplot of community structure. **b** Heapmap showing the top 15 colonies.


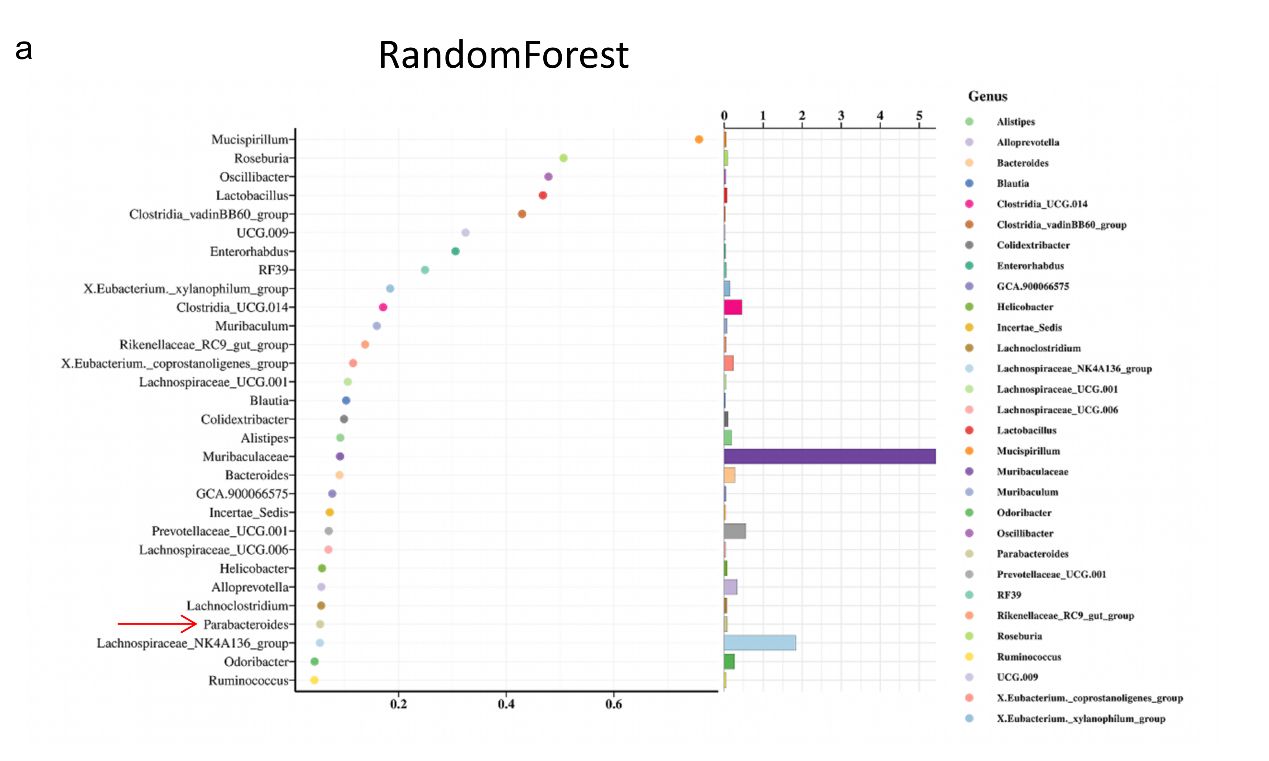


**Fig. S6. Randomforest result showing the import colonies.**


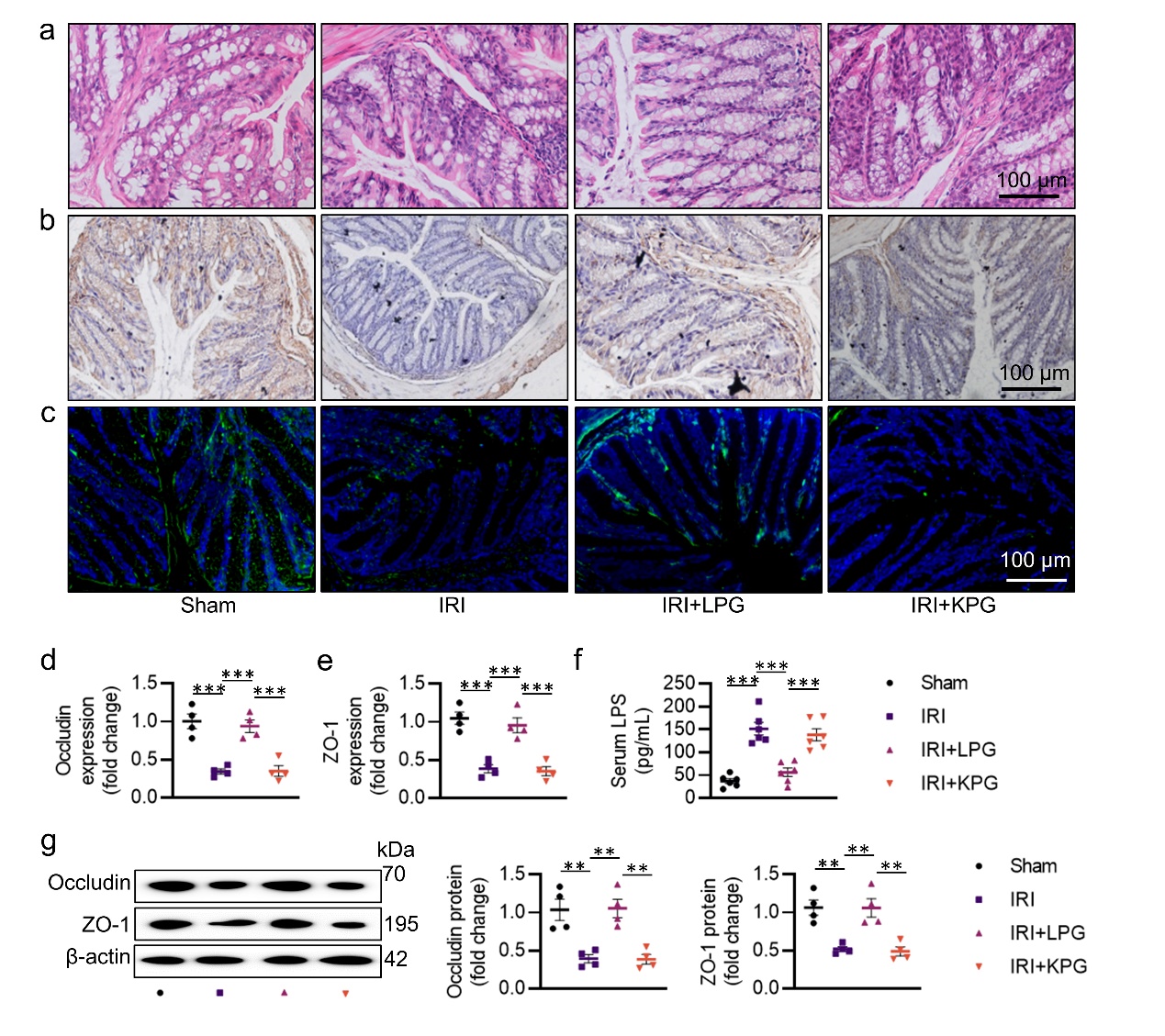


**Fig. S7. The effect of** ***P. goldsteinii* on IR-induced intestinal damage.** **a** Representative photographs H&E staining in colon. **b** Representative immunohistochemistry staining of Occludin in the colon. **c** Representative immunofluorescence staining of ZO-1 in the colon. **d** The injury score of colon. (**e**) Relative analysis of Occludin. **f** Relative analysis of ZO-1. **g** Representative western blots and quantitative analysis of Occludin and ZO-1. *P<0.05, **P<0.01, ***P<0.001 versus the indicated group.


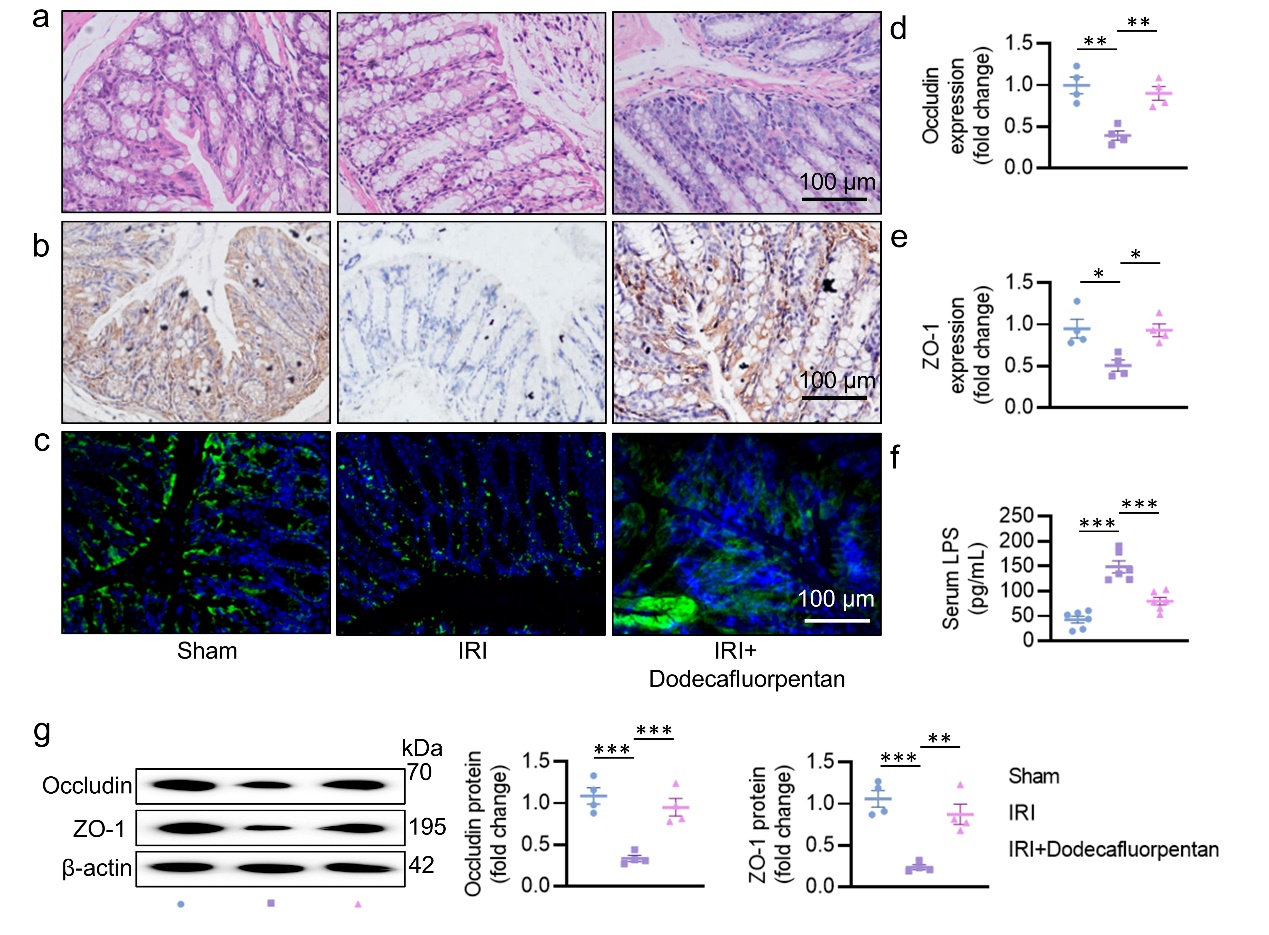


**Fig. S8. The effect of dodecafluorpentan on IR-induced intestinal damage. a** Representative photographs H&E staining in colon. **b** Representative immunohistochemistry staining of Occludin in the colon. **c** Representative immunofluorescence staining of ZO-1 in the colon. **d** Relative analysis of Occludin. **e** Relative analysis of ZO-1. **f** Serum LPS levels. **g** Representative western blots and quantitative analysis of Occludin and ZO-1. *P<0.05, **P<0.01, ***P<0.001 versus the indicated group.


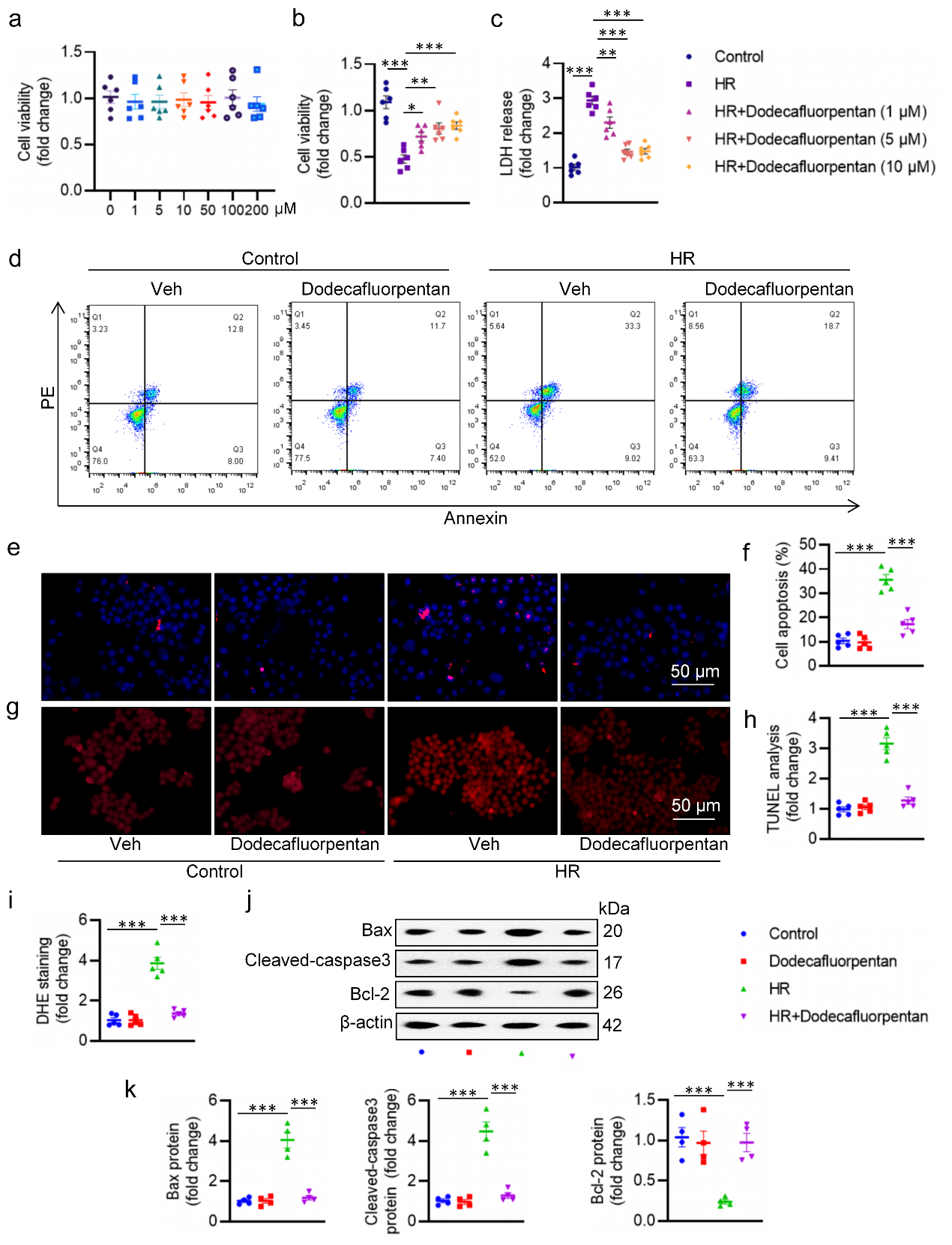


**Fig. S9. The effect of dodecafluorpentan on** **hypoxia/reoxygenation (HR)-induced HK-2 cell damage. a** The cell viability of HK-2 cells at different concentrations of dodecafluorpentan. **b** The cell viability of HK-2 cells induced by HR with or without dodecafluorpentan. **c** The release of LDH. **d** Representative flow cytometry plots of HK-2 cell apoptosis. **e,h** Representative images and quantitative analysis of TUNEL staining (Scale bar = 50 μm). **f** Quantitative analysis of cell apoptosis. **g,i** Representative images and quantitative analysis of DHE staining (Scale bar = 50 μm). **j,k** Representative western blots and quantitative analysis of Bac and Bcl-2. *P<0.05, **P<0.01, ***P<0.001 versus the indicated group.


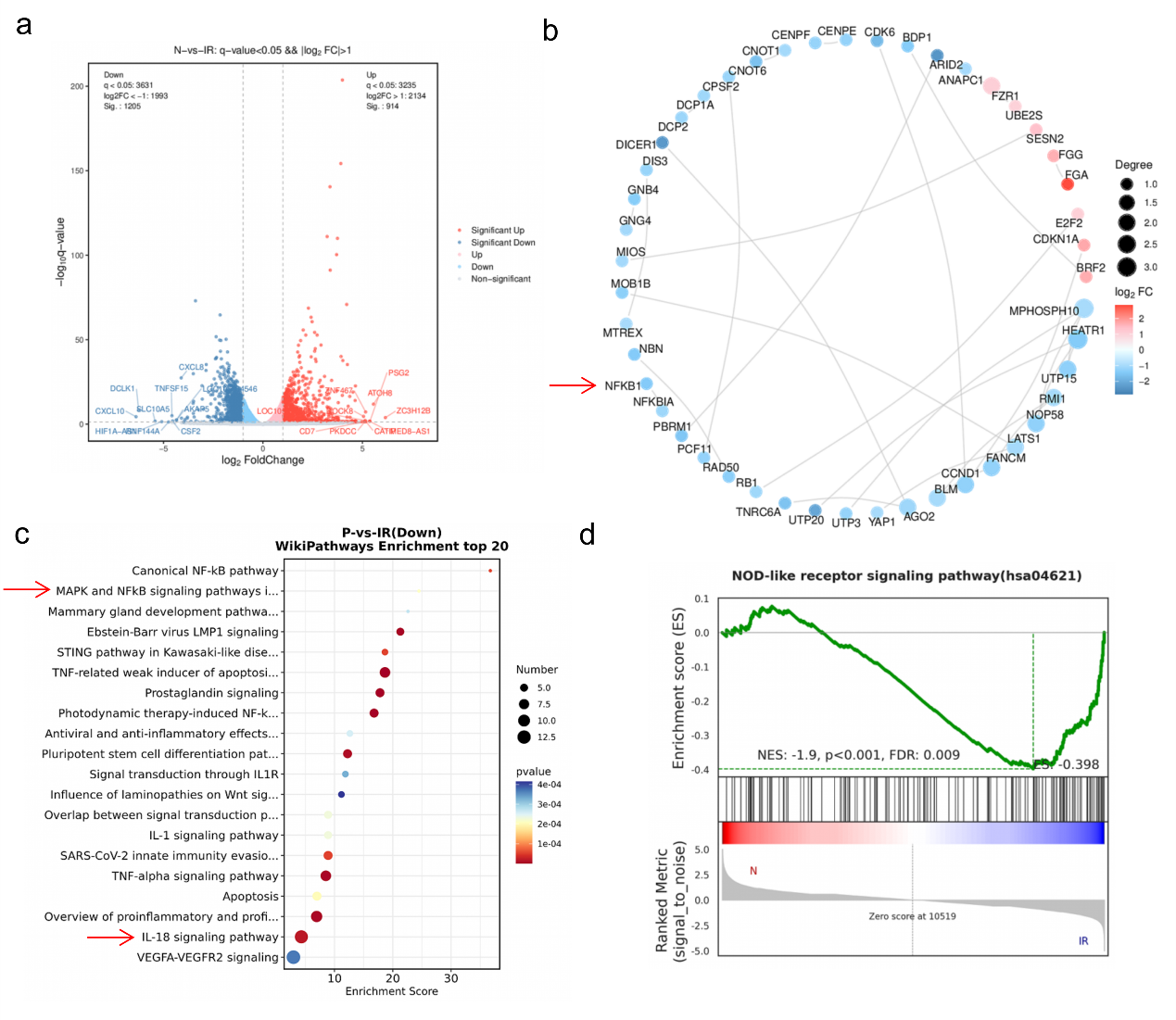


**Fig. S10. Transcriptome of IR and CR-treated IR mice. a** Volcano plot showing the differentially expressed genes in IR and CR-treated IR mice. **b** Protein-protein interaction (PPI) analysis of the identified differentially expressed genes. **c** Gene ontology (GO) pathway enrichment analysis of the differentially expressed genes. **d** Gene set enrichment analysis of NOD-like receptor signaling pathway.


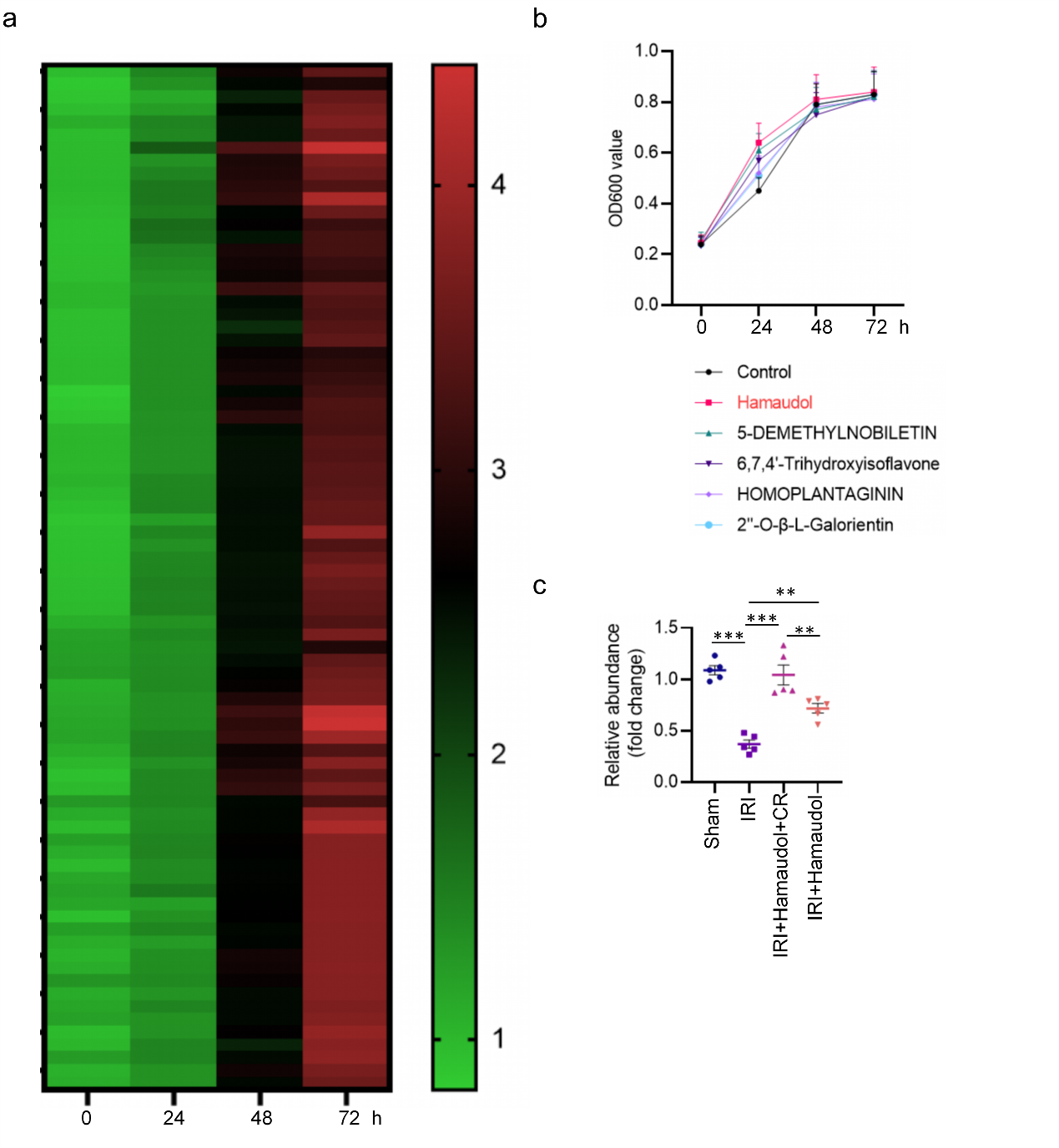


**Figure S11. *In vitro* screening of prebiotics from 79 natural products that promote the growth of *P. goldsteinii.* a** Heatmap showing the effects of different compounds the growth of *P. goldsteinii* at different time points. **b** Top 10 compounds that promoted the growth of *P. goldsteinii* at 24 h. **c** Effects of Hamaudo on the abundance of *P. goldsteinii* in mice. *P<0.05, **P<0.01, ***P<0.001 versus the indicated group.


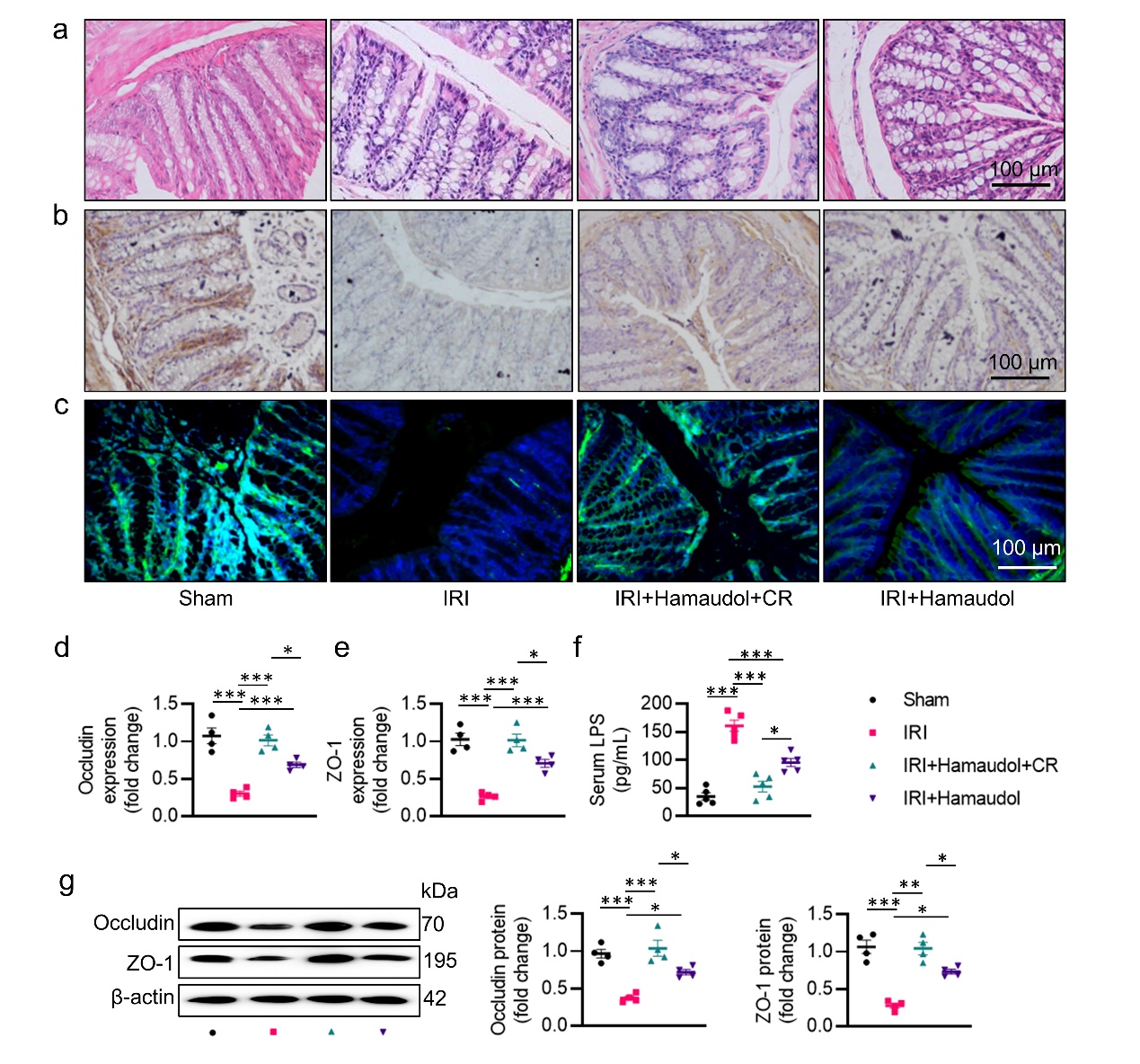


**Figure S12. Hamaudol potentiates the protective effects of CR treatment against IR-induced intestinal injury. a** Representative photographs H&E staining in colon. **b** Representative immunohistochemistry staining of Occludin in the colon. **c** Representative immunofluorescence staining of ZO-1 in the colon. **d** Relative analysis of Occludin. **e** Relative analysis of ZO-1. **f** Serum LPS levels. **g** Representative western blots and quantitative analysis of Occludin and ZO-1. *P<0.05, **P<0.01, ***P<0.001 versus the indicated group.


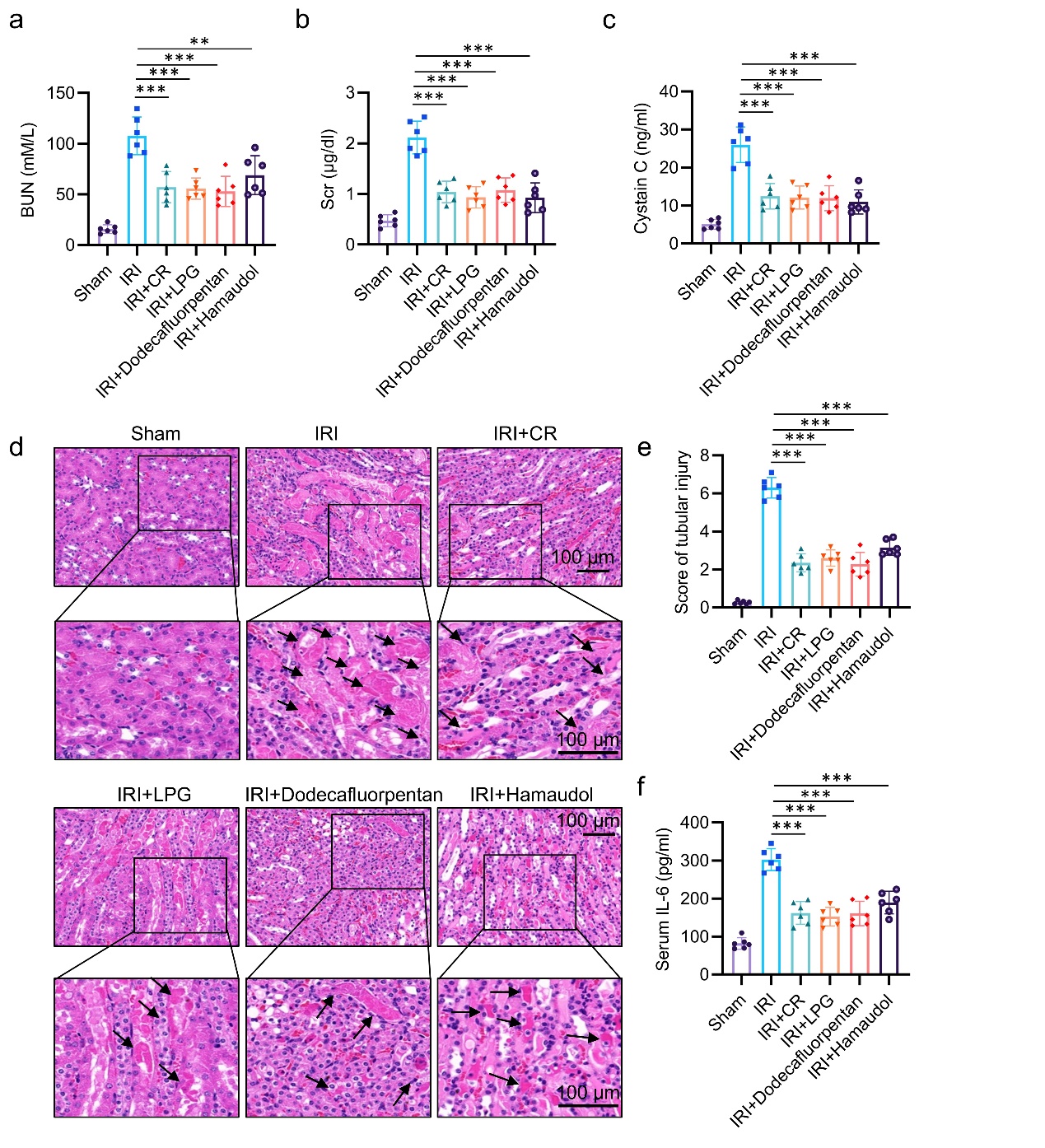


**Figure S13. CR, LPG, dodecafluorpentan, and Hamaudol on renal function after 7 days of renal IRI**. **a** Serum BUN levels. **b** Scr levels. **c** Serum cystatin C levels. **d** Representative HE images of kidney. Arrows indicate injured tubules. **e** Analysis of tubular injury score in mice. **f** The levels of serum IL-6. *P<0.05, **P<0.01, ***P<0.001 versus the indicated group.

**Table S1. The information for different compounds used in this study.**

| **No.** | **Compound** |
| --- | --- |
| 1 | Oroxin A |
| 2 | Procyanidin B1 |
| 3 | Hispidulin |
| 4 | 4',6,7-Trimethoxyisoflavone |
| 5 | Rhamnocitrin |
| 6 | Hamaudol |
| 7 | Gossypin |
| 8 | Dihydrodaidzein |
| 9 | Aromadendrin |
| 10 | 6,7,4'-Trihydroxyisoflavone |
| 11 | 6-Methoxyluteolin |
| 12 | 8-Prenylnaringenin |
| 13 | Sakuranetin |
| 14 | Skullcapflavone II |
| 15 | 4',5,7-Trimethoxyflavone |
| 16 | (±)-Catechin hydrate |
| 17 | 4'-METHOXYFLAVONE |
| 18 | 3,4-Dihydroxyflavone |
| 19 | 5,7,3',4'-Tetramethoxyflavone |
| 20 | Norwogonin |
| 21 | Kaempferol 3-gentiobioside |
| 22 | Visnagin |
| 23 | 5-Hydroxyflavone |
| 24 | Aloeresin D |
| 25 | EGCG Octaacetate |
| 26 | Tilianin |
| 27 | 6-Demethoxytangeretin |
| 28 | Quercetagetin |
| 29 | 4-METHOXYCHALCONE |
| 30 | 7-Hydroxyflavanone |
| 31 | 3-Methoxyflavone |
| 32 | 4-Hydroxyflavanone |
| 33 | 2-Hydroxyflavanone |
| 34 | 3,6-Dihydroxyflavone |
| 35 | 2-Hydroxychalcone |
| 36 | 4'-Hydroxychalcone |
| 37 | 7-Methoxyflavone |
| 38 | Reynoutrin |
| 39 | Theaflavin |
| 40 | 7-Hydroxyflavone |
| 41 | Procyanidin B2 |
| 42 | Apigenin-7-glucuronide |
| 43 | Sophoraflavanone G |
| 44 | Kurarinone |
| 45 | Kaempferol 3-glucorhamnoside |
| 46 | MOSLOFLAVONE |
| 47 | 5,7-DIMETHOXYFLAVONE |
| 48 | 5-hydroxy-7,8-dimethoxyflavone |
| 49 | Vitexia-glucoside |
| 50 | 5-DEMETHYLNOBILETIN |
| 51 | 2''-O-β-L-Galorientin |
| 52 | GALANGIN-3-METHYLETHER |
| 53 | 4',7-DIMETHOXY-5-HYDROXYFLAVONE |
| 54 | Maltol |
| 55 | Cyanidin Chloride |
| 56 | 3’- Methoxy Puerarin |
| 57 | Trifolirhizin |
| 58 | Quercetin-3-O-β-D-glucose-7-O-β-D-gentiobiosiden |
| 59 | HOMOPLANTAGININ |
| 60 | Isoliquiritin apioside |
| 61 | 7,2'-dihydroxy-3',4'-dimethoxyisoflavane-7-O-glucoside |
| 62 | Karanjin |
| 63 | 4-Hydroxycoumarin |
| 64 | Quercetagitrin |
| 65 | Procyanidin C1 |
| 66 | Hesperetin 7-O-glucoside |
| 67 | Iristectorigenin A |
| 68 | Isorhamnetin-3-O-glucoside |
| 69 | Quercimeritrin |
| 70 | Luteolin-3-O-beta-D-glucuronide |
| 71 | Neoeriocitrin |
| 72 | Taxifolin 7-O-rhamnoside |
| 73 | Vicenin 3 |
| 74 | Vaccarin |
| 75 | Glycerol Tri-n-octanoate |
| 76 | Glycerol Trieicosanoate |
| 77 | Glycerol Tridecanoate |
| 78 | Glycerol trilinoleate |
| 79 | 1-Oleoyl-rac-glycerol |

**Table S2. Baseline characteristics for enrolled subjects in this study.**

| Characteristic | Non-AKI (n=30) | AKI (n=30) | P value |
| --- | --- | --- | --- |
| Age (year) | 54.6±2.1 | 56.6±3.1 | 0.633 |
| Male (%) | 17 (56.7) | 19 (63.3) | 0.598 |
| BMI (kg/m^2^) | 22.6±2.2 | 23.1±2.6 | 0.733 |
| BUN (mM) | 5.6±0.3 | 15.8±1.7 | <0.0001 |
| Scr (μM) | 79.9±2.8 | 332.1±40.3 | <0.0001 |

**Table S3. The information for forward and reverse primers used in mice.**

| **Gene** | **Primer sequences** |
| --- | --- |
| IL-1β | Forward: 5’-TTACAGTGGCAATGAGGATG-3’ |
|  | Reverse: 5’-TGTAGTGGTGGTCGGAGATT-3’ |
| IL-6 | Forward: 5’-GCCTTCTTGGGACTGATGCT-3’ |
|  | Reverse: 5’-TGTGACTCCAGCTTATCTCTTGG-3’ |
| MCP-1 | Forward: 5’-TTCTTCGATTTGGGTCTCCTTG-3’ |
|  | Reverse: 5’-GTGCAGCTCTTGTCGGTGAA-3’ |
| COX-2 | Forward: 5’-CACCCTGACATAGACAGTGAAAG-3’ |
|  | Reverse: 5’-CTGGGTCACGTTGGATGAGG-3’ |
| Occludin | Forward: 5’-TGAAAGTCCACCTCCTTACAGA-3’ |
|  | Reverse: 5’-CCGGATAAAAAGAGTACGCTGG-3’ |
| ZO-1 | Forward: 5’-GCCGCTAAGAGCACAGCAA-3’ |
|  | Reverse: 5’-GCCCTCCTTTTAACACATCAGA-3’ |
| β-actin | Forward: 5’-ATGCCCTGAGGCTCTTTTCC-3’ |
|  | Reverse: 5’-CAGCTCAGTAACAGTCCGCC-3’ |

**Table S4. Body weight in mice from different groups after CR intervention.**

|  | **Sham** | **Sham+CR** | **IRI** | **IRI+CR** |
| --- | --- | --- | --- | --- |
| Body weight (g) | 24.2±0.31 | 21.8±0.29* | 24.3±0.17 | 20.65±0.39* |

**Note: Values were expressed as** means ± SEM, *P<0.05 *vs.* Sham.

**Table S5. Body weight in mice from different groups after Abx treatment.**

|  | **IRI** | **IRI+Abx** | **IRI+CR** | **IRI+Abx+CR** |
| --- | --- | --- | --- | --- |
| Body weight (g) | 24.5±0.23 | 24.6±0.24 | 21.9±0.22* | 20.9±0.25* |

**Note: Values were expressed as** means ± SEM, *P<0.05 *vs.* IRI.

**Table S6. Body weight in mice from different groups after FMT treatment.**

|  | **FMT-(Con)** | **FMT-(CR)** |
| --- | --- | --- |
| Body weight (g) | 24.6±0.22 | 24.4±0.26 |

**Note: Values were expressed as** means ± SEM.

**Table S7. Body weight in mice from different groups after *P. goldsteinii* treatment.**

|  | **Sham** | **IRI** | **IRI+LPG** | **IRI+KPG** |
| --- | --- | --- | --- | --- |
| Body weight (g) | 25.1±0.24 | 24.8±0.26 | 25.2±0.24 | 24.1±0.21 |

**Note: Values were expressed as** means ± SEM.

**Table S8. The differentially expressed metabolites.**

| **No.** | **Ion mode** | **Metabolites** |
| --- | --- | --- |
| 1 | neg | PE(DiMe(13,5)/20:3(5Z,11Z,14Z)-O(8,9)) |
| 2 | neg | PE-NMe(20:4(8Z,11Z,14Z,17Z)/20:0) |
| 3 | neg | PE(22:4(7Z,10Z,13Z,16Z)/19:0) |
| 4 | neg | Dodecafluoropentane |
| 5 | neg | 3,5-Dihydroxytetradecanoylcarnitine |
| 6 | neg | PE-NMe2(18:3(9Z,12Z,15Z)/18:1(11Z)) |
| 7 | neg | TETRANITROMETHANE |
| 8 | pos | Acetylcarnosine |
| 9 | neg | Fucoxanthinol 3-myristoleate |
| 10 | pos | 3-deoxy-D-manno-octulosonate |
| 11 | neg | alpha-D-xylose 1-phosphate |
| 12 | neg | CDP-DG(TXB2/20:3(5Z,8Z,11Z)) |
| 13 | neg | PS(P-16:0/19:0) |
| 14 | neg | PS(PGJ2/22:1(13Z)) |
| 15 | neg | PE-NMe(20:4(8Z,11Z,14Z,17Z)/18:0) |
| 16 | pos | PE(18:2(9Z,12Z)/P-18:1(11Z)) |
| 17 | neg | PC(16:1(9Z)/17:1(9Z)) |
| 18 | pos | PE(19:1(9Z)/15:0) |
| 19 | pos | Sphingosine |
| 20 | neg | PS(22:2(13Z,16Z)/PGJ2) |
| 21 | neg | PE-NMe2(22:1(13Z)/14:1(9Z)) |
| 22 | pos | SM(d16:2(4E,8Z)/PGF1alpha) |
| 23 | pos | PC(P-16:0/18:1(9Z)) |
| 24 | pos | PE(O-20:0/15:0) |
| 25 | neg | Uric acid |
| 26 | neg | Thiophene, 2-nitroso- |
| 27 | pos | Palmitoylcarnitine |
| 28 | pos | 11-Hydroxyhexadecanoylcarnitine |
| 29 | pos | L-Phenylalaninamide,L-prolyl-L-histidyl-L-prolyl-L-phenylalanyl-L-histidyl-(3S,4S)-  4-amino-3-hydroxy-6-methylheptanoyl-L-isoleucyl-(9CI) |
| 30 | neg | Octadec-9-enoic Acid |
| 31 | pos | PE(16:1(9Z)/20:3(5Z,8Z,11Z)) |
| 32 | pos | GM4(d18:1/20:0) |
| 33 | pos | PA(20:3(5Z,8Z,11Z)/18:0) |
| 34 | pos | LysoPE(0:0/20:4(8Z,11Z,14Z,17Z)) |
| 35 | pos | LysoPE(20:5(5Z,8Z,11Z,14Z,17Z)/0:0) |
| 36 | neg | 3,4-Dihydroxyphenylvaleric acid 4 sulfate |
| 37 | neg | 21-Deoxycortisol |
| 38 | pos | Tetrahydroaldosterone |
| 39 | pos | Pent-1-en-2-ol |
| 40 | pos | SM(d18:0/5-iso PGF2VI) |
| 41 | pos | PE(20:3(8Z,11Z,14Z)/18:0) |
| 42 | pos | LysoPE(0:0/22:6(4Z,7Z,10Z,13Z,16Z,19Z)) |
| 43 | pos | DG(18:0/20:4(8Z,11Z,14Z,17Z)/0:0) |
| 44 | pos | 2-Lysophosphatidylcholine |
| 45 | pos | PI(22:0/14:1(9Z)) |
| 46 | pos | L-Carnitine |
| 47 | neg | alpha-Methyl-m-tyrosine |
| 48 | neg | ribostamycin |
| 49 | neg | 6,7-Dimethoxy-2-(piperazin-1-yl)quinazolin-4-amine |
| 50 | neg | 4-Nitro-o-phenylenediamine |
| 51 | neg | (±)-2-(1-Methylpropyl)-4,6-dinitrophenol |
| 52 | neg | 1-(2-Furanyl)-1-butanone |
| 53 | neg | Alepramic acid |
| 54 | neg | (2R,3S)-2,3-dimethylmalate |
| 55 | neg | 6-Hydroxynicotinamide |
| 56 | neg | Pretyrosine |
| 57 | neg | N-acetyl-L-2-aminoadipate(2-) |
| 58 | neg | (Z)-3-(1-Formyl-1-propenyl)pentanedioic acid |
| 59 | neg | 4-Amino-5-hydroxy-1-[(2R,5R)-4-hydroxy-5-(hydroxymethyl)oxolan-2-yl]pyrimidin-2-one |
| 60 | neg | 6-Hydroxymethylpterin |
| 61 | neg | (R)-Dihydromaleimide |
| 62 | neg | 5-Methyl-2(3H)-furanone |
| 63 | neg | A-Ketoglutaric acid oxime |
| 64 | neg | 2-Iminiopropanoate |
| 65 | neg | p-Benzosemiquinone |
| 66 | neg | Telbivudine |
| 67 | neg | N-(Hydroxymethyl)acrylamide |
| 68 | neg | Pyrrolidino-[1,2E]-4H-2,4-dimethyl-1,3,5-dithiazine |
| 69 | neg | 2-aceto-2-hydroxy-butanoate |
| 70 | neg | Fluoxetine |
| 71 | neg | Idazoxan |
| 72 | neg | 5-Dihydroxyhept-6-enoic acid |
| 73 | neg | 3-Methoxytyrosine |
| 74 | neg | Galantamine |
| 75 | neg | Butanoic acid, 2,3-dihydroxypropyl ester |
| 76 | neg | 1,2-Dioxolane |
| 77 | neg | xi-2,3-Dihydro-3,5-dihydroxy-6-methyl-4H-pyran-4-one |
| 78 | neg | Phenylalanylphenylalanine |
| 79 | neg | 4-Hydroxy Triamterene |
| 80 | neg | (2S,2'S)-Pyrosaccharopine |
| 81 | neg | 6-Methyl-N-tetrazol-5-yl-2-pyridinecarboxamide |
| 82 | neg | D-1,5-Anhydrofructose |
| 83 | neg | (1R,3S)-3-(6-Aminopurin-9-yl)cyclopentan-1-ol |
| 84 | neg | Ethyl nicotinate |
| 85 | neg | voxtalisib |
| 86 | neg | 2,3,4,5-Tetrahydro-2-pyridinecarboxylic acid |
| 87 | neg | 6-isobutyl-4-hydroxy-2-pyrone |
| 88 | neg | 2,4(1H,3H)-Pyridinedione |
| 89 | neg | Hexahydropyrrolo[1,2-a]pyrazine-1,4-dione |
| 90 | neg | Pi-Methylimidazoleacetic acid |
| 91 | neg | MALONONITRILE |
| 92 | neg | Descyclopropyl Abacavir |
| 93 | neg | indole-3-acetyl-leucine |
| 94 | neg | Glutaminylhydroxyproline |
| 95 | neg | 5H-Pyrrolo[3,2-d]pyrimidine |
| 96 | neg | Hydroxyprolylhydroxyproline |
| 97 | neg | Fructosamine |
| 98 | neg | 9-(Tetrahydrofuran-2-yl)-9h-purin-6-amine |
| 99 | neg | (S)-Spinacine |
| 100 | neg | Triamterene |
| 101 | neg | 4,6-O-Ethylidene-alpha-D-glucose |
| 102 | neg | Ethyl oxyhydrate |
| 103 | pos | 2-Linoleoyl Glycerol |
| 104 | neg | LysoPE(0:0/18:1(11Z)) |
| 105 | neg | 1-O-Hexadecyl-sn-glycero-3-phosphocholine |
| 106 | neg | Retaspimycin |
| 107 | pos | 3-Amino-3-methylbutanoic acid |
| 108 | pos | L-Lysine |
| 109 | neg | PS(20:4(5Z,8Z,11Z,14Z)/0:0) |
| 110 | neg | OKHdiA-PE |
| 111 | pos | Phe-Phe-Pro-Arg |
| 112 | neg | Foliandrin |
| 113 | neg | 17-AAG |
| 114 | pos | 3-Methyl sulfolene |
| 115 | neg | Eicosatetraenoic acid |
| 116 | neg | PE(O-18:0/0:0) |
| 117 | pos | Statine |
| 118 | neg | 2-Phenylethanol glucuronide |
| 119 | neg | Thiophene-4,5-epoxide |
| 120 | neg | Sodium Tetradecyl Sulfate |
| 121 | pos | (4E,15E)-Bilirubin |
| 122 | neg | Berkeleylactone M |
| 123 | neg | (3b,6b,8a,12a)-8,12-Epoxy-7(11)-eremophilene-6,8,12-trimethoxy-3-ol |
| 124 | neg | 2-Pyrocatechuic acid |
| 125 | neg | Glucose lactate ketone |
| 126 | pos | (2E,4E)-2,4-Nonadien-1-ol |
| 127 | neg | 2,5-Dihydroxybenzenesulfonic Acid |
| 128 | neg | Amobarbital |
| 129 | neg | Bromelains |
| 130 | neg | Thiolanilide |
| 131 | pos | hydroxynonenal |
| 132 | pos | PIP(PGF1alpha/22:2(13Z,16Z)) |
| 133 | neg | Saccharopine |
| 134 | pos | 1-(2-methoxy-hexadecanyl)-sn-glycero-3-phosphoserine |
| 135 | pos | Sphinganine |
| 136 | neg | C75 trans |
| 137 | pos | Cladosporacid B |
| 138 | neg | PIP(22:3(10Z,13Z,16Z)/20:4(6E,8Z,11Z,14Z)-OH(5S)) |
| 139 | neg | 2-Hydroxyundecan-6-one |
| 140 | pos | Aspartame |
| 141 | neg | 3',5'-Dideoxythymidine |
| 142 | neg | Glaucarubin |
| 143 | neg | 6-Deoxypenciclovir |
| 144 | neg | N-(1-Deoxy-1-fructosyl)tyrosine |
| 145 | neg | rac S 33138 |
| 146 | neg | Tyrosyl-alanyl-glycine |
| 147 | pos | Neoconvallatoxoloside |
| 148 | neg | 2-Ethylpropanedioylcarnitine |
| 149 | neg | N,N-Diallyl-tyrosyl-aminoisobutyryl-aminoisobutyryl-phenylalanyl-leucine |
| 150 | pos | 26-O-beta-d-glucopyranosyl-(22S,25S)-furostan-22,25-  epoxy-3beta,5beta,26,27-tetrol-5-O-beta-d-glucopyranoside |

**Table S9. Body weight in mice from different groups after dodecafluorpentan treatment.**

|  | **Sham** | **IRI** | **IRI+ dodecafluorpentan** |
| --- | --- | --- | --- |
| Body weight (g) | 25.4±0.23 | 24.9±0.24 | 25.3±0.31 |

**Note: Values were expressed as** means ± SEM.

**Table S10. Body weight in mice from different groups after Hamaudol intervention.**

|  | **Sham** | **IRI** | **IRI+ Hamaudol+CR** | **IRI+Hamaudol** |
| --- | --- | --- | --- | --- |
| Body weight (g) | 24.6±0.27 | 24.8±0.31 | 21.9±0.19* | 25.1±0.28 |

**Note: Values were expressed as** means ± SEM, *P<0.05 *vs.* Sham.
